# PIP_3_-Phldb2 is crucial for LTP regulating synaptic NMDA and AMPA receptor density and PSD95 turnover

**DOI:** 10.1038/s41598-019-40838-6

**Published:** 2019-03-13

**Authors:** Min-Jue Xie, Yasuyuki Ishikawa, Hideshi Yagi, Tokuichi Iguchi, Yuichiro Oka, Kazuki Kuroda, Keiko Iwata, Hiroshi Kiyonari, Shinji Matsuda, Hideo Matsuzaki, Michisuke Yuzaki, Yugo Fukazawa, Makoto Sato

**Affiliations:** 1 Division of Cell Biology and Neuroscience, Department of Morphological and Physiological Sciences, Faculty of Medical Sciences, University of Fukui, Fukui, 910-1193 Japan; 2 Division of Brain Structures and Function, Department of Morphological and Physiological Sciences, Faculty of Medical Sciences, University of Fukui, Fukui, 910-1193 Japan; 3 Division of Development of Mental Functions, Research Centre for Child Mental Development, University of Fukui, Fukui, 910-1193 Japan; 4 Life Science Innovation Centre, University of Fukui, Fukui, 910-1193 Japan; 5 United Graduate School of Child Development, Osaka University, Kanazawa University, Hamamatsu University School of Medicine, Chiba University and University of Fukui, Osaka University, Osaka, 565-0871 Japan; 6 Department of Systems Life Engineering, Maebashi Institute of Technology, Gunma, 371-0816 Japan; 7 Laboratory of Functional Neuroscience, Nara Institute of Science and Technology, 8916-5, Takayama, Ikoma, Nara, 630-0192 Japan; 8 Department of Cell Biology, Hyogo College of Medicine, Hyogo, 663-8501 Japan; 9 Department of Anatomy and Neuroscience, Graduate School of Medicine, Osaka University, Osaka, 565-0871 Japan; 10 Animal Resource Development Unit and Genetic Engineering Team, RIKEN Center for Life Science Technologies, Kobe, 650-0047 Japan; 11 Department of Neurophysiology School of Medicine, Keio University, Tokyo, 160-8582 Japan; 12 Department of Engineering Science, Graduate School of Informatics and Engineering, University of Electro-Communications, Tokyo, 182-8585 Japan; 13 Japan Science and Technology Agency, PRESTO, Saitama, 332-0012 Japan

## Abstract

The essential involvement of phosphoinositides in synaptic plasticity is well-established, but incomplete knowledge of the downstream molecular entities prevents us from understanding their signalling cascades completely. Here, we determined that Phldb2, of which pleckstrin-homology domain is highly sensitive to PIP_3_, functions as a phosphoinositide-signalling mediator for synaptic plasticity. BDNF application caused Phldb2 recruitment toward postsynaptic membrane in dendritic spines, whereas PI3K inhibition resulted in its reduced accumulation. Phldb2 bound to postsynaptic scaffolding molecule PSD-95 and was crucial for localization and turnover of PSD-95 in the spine. Phldb2 also bound to GluA1 and GluA2. Phldb2 was indispensable for the interaction between NMDA receptors and CaMKII, and the synaptic density of AMPA receptors. Therefore, PIP_3_-responsive Phldb2 is pivotal for induction and maintenance of LTP. Memory formation was impaired in our *Phldb2*^−/−^ mice.

## Introduction

Long-lasting changes in the strength of synaptic transmission, such as long-term potentiation (LTP)^1^ and long-term depression (LTD)^2^, underlies learning and memory. Glutamate, which is released from the presynaptic terminals, activates postsynaptic N-methyl-D-aspartate-type glutamate receptors (NMDA receptors) and α-amino-3-hydroxy-5-methyl-4-isoxazole propionic acid-type glutamate receptors (AMPA receptors)^3,4^. High-frequency presynaptic stimulation induces the opening of NMDA receptors and subsequent calcium entry into the cell, leading to LTP induction^5^. LTP in the hippocampus is intensively studied and has been characterized as follows: high-frequency stimulation of the Schaffer collaterals leads to LTP induction in the pyramidal neurons of CA1. The resultant elevation of intracellular calcium concentration triggers a biochemical cascade that includes the activation of calcium/calmodulin-dependent protein kinase II (CaMKII) and eventually leads to the long-lasting potentiation of AMPA receptor-mediated excitatory postsynaptic currents (EPSCs). These are underlying molecular machineries for learning and memory^5,6^.

Phosphatidylinositol 3,4,5-triphosphate (PIP_3_), one of the phosphoinositides, plays important roles in a diverse range of cellular functions as a lipid second messenger. After stimulation of the cell by a chemoattractant, phosphatidylinositol 3-kinase (PI3K) is locally activated, resulting in the transient accumulation of PIP_3_ on the leading edge of directed migrating amoebas and leukocytes^7^. PIP_3_ works, at least in part, as a cell compass that translates external signals into directed cell movement^8^. In neurons, it has been shown that PIP_3_ is concentrated at the dendritic spines, regulates spinule formation and is crucial for maintaining AMPA receptor clustering during LTP^9,10^. Furthermore, brain-derived neurotrophic factor (BDNF) and its receptor trkB, for which PIP_3_ works, have attracted much attention for synaptic plasticity^11–13^. It is not clear what molecule mediates the relationship between PIP_3_ and the AMPA receptor during synaptic plasticity.

Proteins involved in signal transduction and cytoskeletal dynamics interact with membrane phosphoinositides through their pleckstrin-homology (PH) domains, and phosphoinositides act, in large part, through such proteins^14^. Although the core tertiary structure is conserved among PH domains, high sequence variability among their primary structures (approximately 120 amino acids) gives rise to different specificities of PH domains for different phosphoinositides^15^. Phldb2 (pleckstrin homology-like domain, family B, member 2, alternatively called LL5β) is a protein of approximately 160 kDa that contains two predicted coiled-coil domains and a PH domain whose binding is highly sensitive to PIP_3_ as well as PIP_2_^16^. In our previous report, we showed that Phldb2 helps its binding partner, filamin A, translocate to the leading edge of the plasma membrane where PIP_3_ is accumulated in migrating cells^17^. By sensing PIP_3_, Phldb2 is likely to work as a hub for subsequent intracellular events.

Herein, we demonstrate that Phldb2 localizes in the dendritic spines of the hippocampal neurons, functions as a phosphoinositide-responsive entity and plays a pivotal role in synaptic plasticity.

## Results

### Phldb2 co-localizes with PSD-95 in the spines of hippocampal neurons

Phldb2 mRNA was expressed in the hippocampus; some neurons strongly expressed Phldb2 mRNA and the expression of Phldb2 was confirmed with our Phldb2 antibody (Supplementary Fig. 1A–C). In this study, we investigated the *in vivo* roles of Phldb2 in the hippocampus. Biochemically, Phldb2 was enriched in the synaptosomal fraction and the synaptic membrane fraction (Supplementary Fig. 1D). Immunocytochemically, Phldb2 was observed in the spines of the hippocampal neurons, co-localizing well with the postsynaptic scaffold protein PSD-95 (Supplementary Fig. 1E,F), which is a major component of the postsynaptic density and regulates the maturation of dendritic spines.

### PIP_3_ plays a critical role in the precise localization of Phldb2

**Figure 1:**
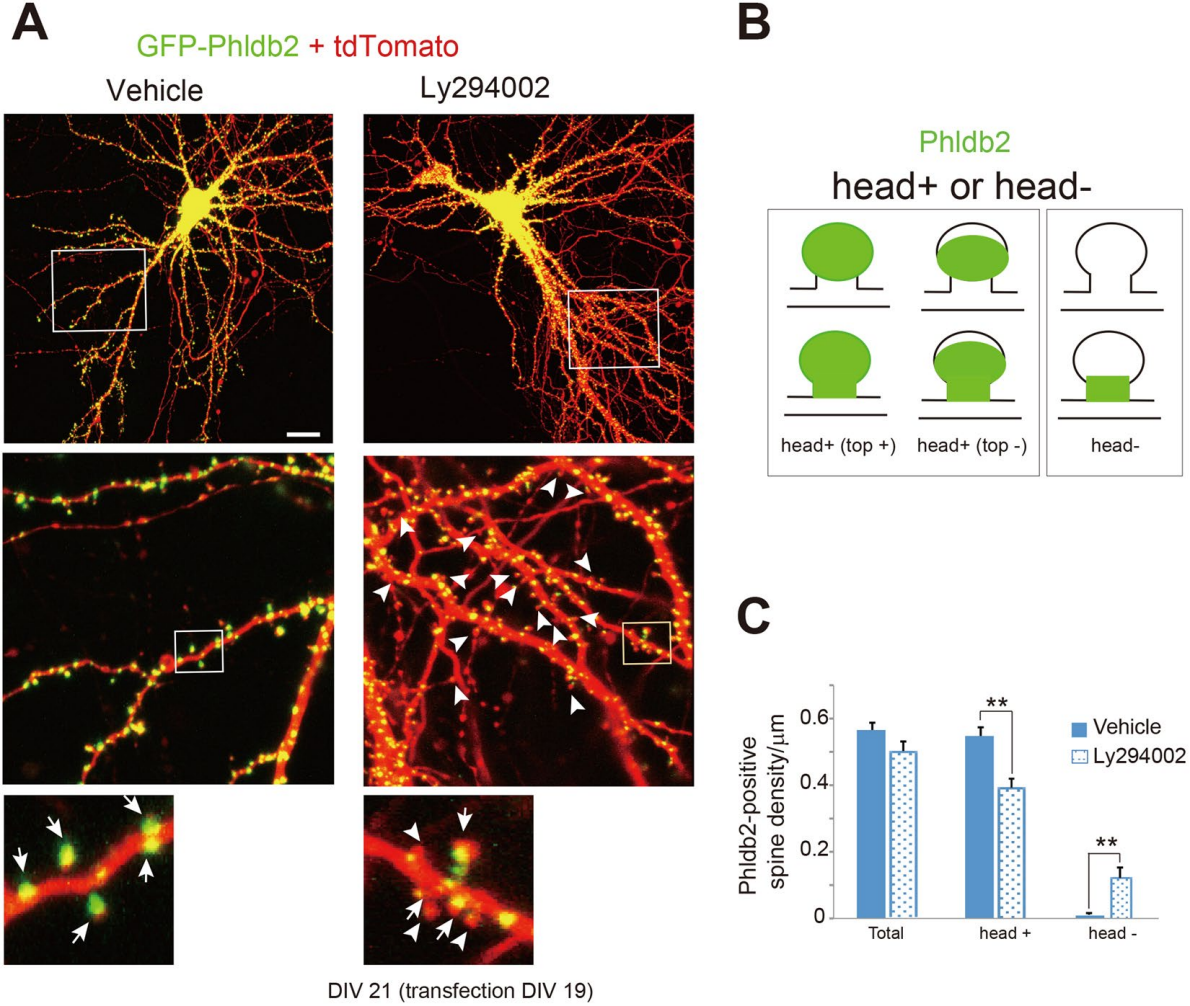
A PI3K inhibitor induces a decrease in the number of spines with Phldb2-positive heads. (**A**) Cultured hippocampal neurons were cotransfected with expression vectors for GFP-Phldb2 and tdTomato. GFP-Phldb2 localized in the dendritic spines. Neurons subjected to 10 μM Ly294002 treatment for 1 hr are shown in the right panels. Representative neurons are shown in the upper panels. Magnified images of the squares are shown in the panels below. (**B**) Head+ indicates a spine whose head is Phldb2 positive, whereas head- indicates a spine whose head is Phldb2 negative. Typical examples are shown in (**A**) Head+ spines are shown by arrows, whereas head- spines are indicated by arrowheads. (**C**) The densities of head+ and head- spines were measured in secondary and tertiary dendrites. GFP-Phldb2-transfected neurons were treated with Ly294002 (Ly294002, n = 322 spines) or did not receive Ly294002 treatment (vehicle, n = 686 spines) (Mean ± SEM. Student’s *t*-test, **P < 0.01). Scale bar represents 20 μm in (**A**).

Since Phldb2 has a PH domain, which has a particularly high affinity for PIP_3_^16^, we asked whether PIP_3_ regulates Phldb2 localization in the dendritic spines. It has been shown that PIP_3_ plays a role in the spines^9,10^. We observed that Phldb2 tagged with green fluorescent protein at its N-terminus (GFP-Phldb2) was enriched in the spines (Fig. 1A). However, GFP-Phldb2 showed less accumulation in the spine when the PI3K inhibitor Ly294002 was applied (Fig. 1A). We confirmed this observation by categorizing spines as ‘head+’ or ‘head−’, based on the subcellular localization of Phldb2, and counting the number of spines in each category (Fig. 1B). Almost all the spines (approximately 98%) fell into the ‘head+’ category in the absence of treatment, but the proportion of ‘head+’ spines decreased after Ly294002 treatment (Fig. 1C). Spine density did not significantly change in response to Ly294002 treatment (Fig. 1C). This finding suggests that PIP_3_ is involved in regulating the precise subcellular localization of Phldb2 in the spines.

### Generation of Phldb2 knockout mice

To elucidate the physiological role of Phldb2, we generated Phldb2 knockout mice (*Phldb2*^−/−^ mice) by replacing exon2 (1599 bp including the coding region for amino acids 1 to 441) of the murine Phldb2 gene with floxed exon2 and neo cassette (Supplementary Fig. 2A,B). First, we generated the mice with the targeted allele (*Phldb2*^+/*exon2-neo*^ mice) (Supplementary Fig. 2C), and then we crossed the *Phldb2*^+/*exon2-neo*^ mice carrying a floxed allele with TNAP (tissue-nonspecific alkaline phosphatase)-*Cre* knock-in mice, disrupting Phldb2 in primordial germ cells^18^. The disruption of the gene and the lack of Phldb2 protein expression were then confirmed (Supplementary Fig. 2D–G). Homozygous *Phldb2*^−/−^ mice were born in good health and grew to adulthood.

### Phldb2 regulates the localization of PSD-95 and its turnover in the spines

**Figure 2:**
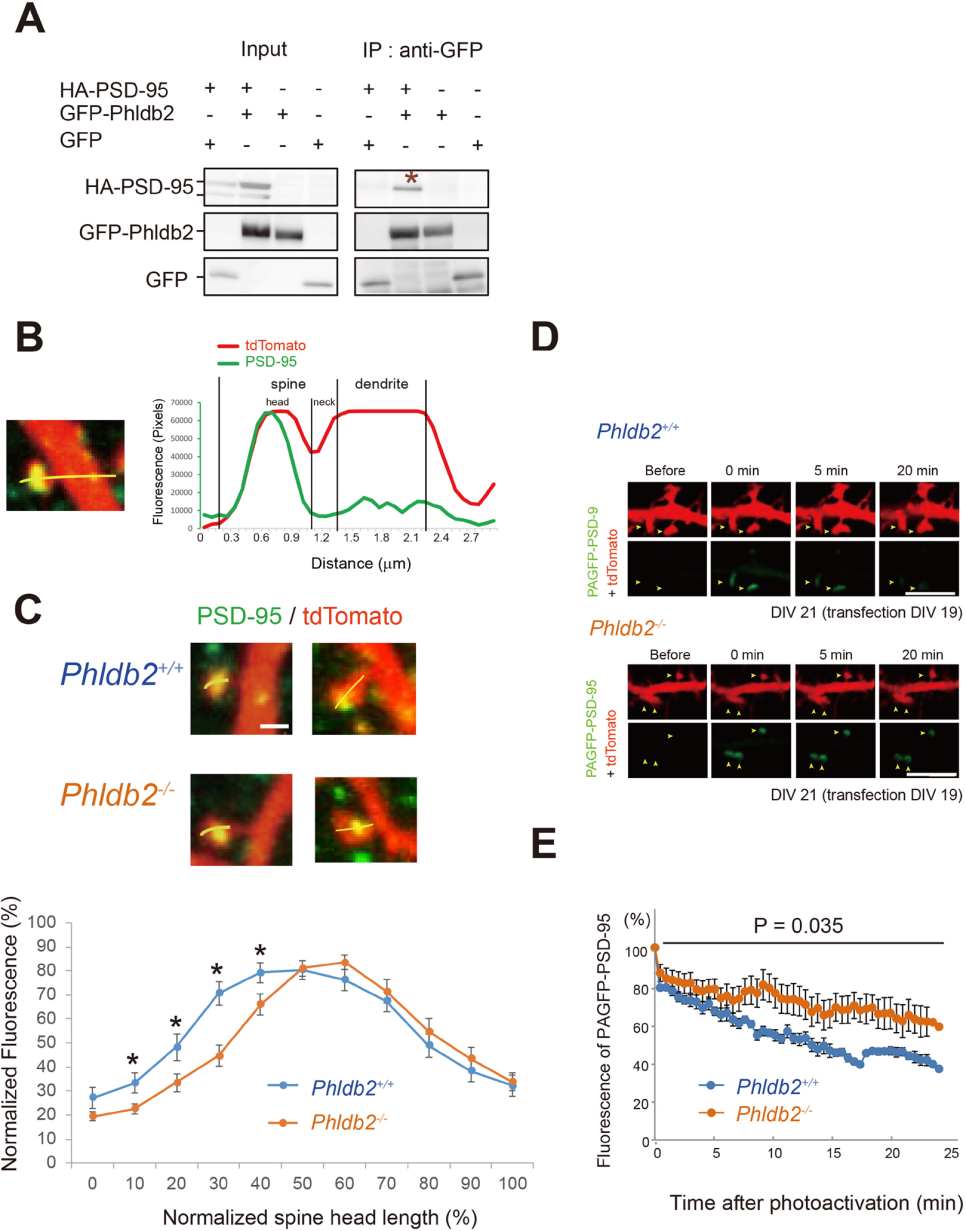
Phldb2 interacts with PSD-95 and regulates PSD-95 turnover. (**A**) Expression vectors for HA-PSD-95 and GFP-Phldb2 or GFP-mock were cotransfected in COS-7 cells. Lysates were subjected to immunoprecipitation with anti-GFP antibody. PSD-95 was co-immunoprecipitated with Phldb2 (star). The grouping of blots was cropped from Supplementary Fig. 3. (**B**) Hippocampal neurons were transfected with tdTomato expression vectors and fixed at day 21 *in vitro* (DIV 21). High-magnification image of a mushroom spine. Endogenous PSD-95 was stained (green). A plot of the fluorescence intensity profile along the yellow line shows the distribution of endogenous PSD-95 (green line) and tdTomato (red line). The spine lengths were defined by the fluorescence intensity profile of tdTomato. (**C**) The spine head lengths along the yellow line were divided into 10 parts, shown on the X-axis. The fluorescence peak of PSD-95 was consistently slightly farther from the spine head in the *Phldb2*^−/−^ mice (n = 23 spines) than in the *Phldb2*^+/+^ mice (n = 25 spines). The Y-axis represents normalized fluorescence intensity. For normalization, signal intensity in the dendritic spine head length was divided by peak intensity (intensity/peak intensity ratio) (Mean ± SEM. Student’s *t*-test, *P < 0.05). (**D**) Cultured hippocampal neurons were cotransfected with expression vectors for photoactivatable green fluorescent protein-tagged PSD-95 (PAGFP-PSD-95) and tdTomato in the *Phldb2*^+/+^ mice and the *Phldb2*^−/−^ mice at DIV 20. PAGFP-PSD-95 was photoactivated by two-photon excitation with 730 nm laser light in the indicated areas (arrowheads) at time 0. (**E**) Semi-quantification of PAGFP-PSD-95 fluorescence in the spine. The rate of fluorescence of intensity of PAGFP-PSD-95 increased in the *Phldb2*^−/−^ mice (*Phldb2*^+/+^ mice, n = 10 spines; *Phldb2*^−/−^ mice, n = 8 spines. Mean ± SEM. Two-way repeated measures ANOVA, F _(1,17)_ = 5.25, *P* = 0.035). Scale bars represent 5 μm.

Since Phldb2 co-localized well with PSD-95 (Supplementary Fig. 1E,F), we asked whether Phldb2 binds to PSD-95. After confirming the binding of Phldb2 to PSD-95 by co-immunoprecipitation (Fig. 2A, Supplementary Figs 3, 4A), we then asked whether Phldb2 regulates the localization of PSD-95 in the spines. The distribution of PSD-95 throughout the spine was measured and analysed (Fig. 2B). PSD-95 location was eccentrically shifted towards the dendritic shaft in the *Phldb2*^−/−^ mice compared with the *Phldb2*^+/+^ mice (Fig. 2C), suggesting that Phldb2 helps PSD-95 localize in the spine head. Exogenous Phldb2 expression rescued the PSD-95 localization in the *Phldb2*^−/−^ mice (Supplementary Fig. 4B). We next used photoactivatable green fluorescent protein-tagged PSD-95 (PAGFP-PSD-95) to investigate whether Phldb2 is involved in the turnover of PSD-95 in the spines (Fig. 2D,E). The fluorescence intensity of photoactivated PAGFP-PSD-95 was much lower in the *Phldb2*^+/+^ mice than in the *Phldb2*^−/−^ mice (Fig. 2E). Therefore, it is likely that Phldb2 maintains the turnover of PSD-95 in the spines. PSD-95 dislocation due to the absence of Phldb2 may modify the turnover, yet how these events interfere with each other remains elusive.

### Phldb2 moves into the spines in response to BDNF

BDNF is a major trophic factor that induces an elevation of PIP_3_ in the spines^19,20^ and plays a crucial role in LTP maintenance^21^. We asked whether Phldb2 functions in the downstream part of BDNF signalling cascades via PIP_3_. Exogenous GFP-tagged Phldb2 accumulated in the spines in response to BDNF in the *Phldb2*^−/−^ mice and disappeared after washout (Supplementary Fig. 5A). In this experiment, we used B27 free medium, since B27 contains some insulin, which may have the similar effects of BDNF^22^. Together with results shown in Supplementary Fig. 5B, Phldb2 localization is regulated by PIP_3_ concentration in the spines.

### Phldb2 is involved in synaptic accumulation of NMDA receptors in the dendritic spines

**Figure 3:**
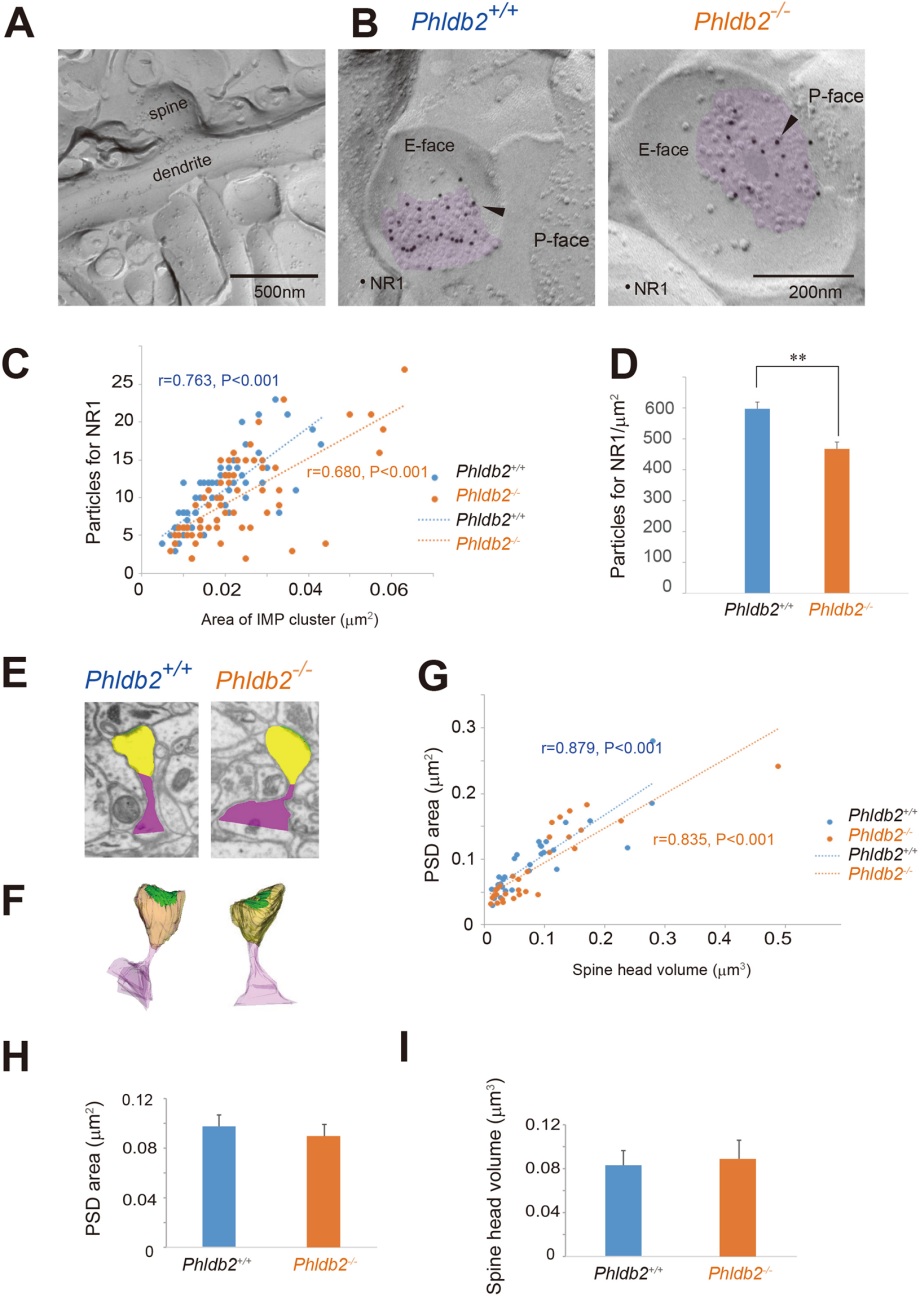
Phldb2 gene deletion results in a significant decrease in synaptic NMDA receptor density without changes in the size of postsynaptic densities or the volume of spine heads. (**A**) Replicas were prepared from the CA1 region of hippocampus, and dendrites and dendritic spines in replicas were identified based on morphology under a transmission electron microscope. (**B**) Postsynaptic membrane specializations of excitatory synapses in replicas were identified in the exoplasmic (E)-face of the plasma membrane by clusters of intra-membrane particles (IMP clusters, purple) labelled for the NR1 subunit (arrowhead) and visualized with 10 nm immunogold. (**C**) The numbers of immunoparticles for NR1 in individual IMP clusters were plotted against the areas of IMP clusters. A statistically significant positive correlation between the NR1 labelling number and the synaptic area was found regardless of genotype (Pearson’s correlation test: the *Phldb2*^+/+^ mice, n = 62 synapses, r = 0.680, *P* < 0.001; the *Phldb2*^−/−^ mice, n = 62 synapses, r = 0.763, *P* < 0.001). (**D**) The average labelling density for synaptic NR1 was significantly lower in the *Phldb2*^−/−^ mice than in the *Phldb2*^+/+^ mice (Mean ± SEM. Student’s *t*-test, **P < 0.001). (**E**,**F**) Reconstruction of dendritic spines from serial FIB-SEM images clearly demonstrates a full view of a dendritic spine, its head portion, and a postsynaptic membrane specialization (green lines) defined by the postsynaptic density (PSD). (**E**) Examples of FIB-SEM images from *Phldb2*^+/+^ mice and *Phldb2*^−/−^ mice (spine head in yellow, PSD in green and the rest of the spine in purple). (**F**) Examples of 3D-reconstructed spines from *Phldb2*^+/+^ and *Phldb2*^−/−^ mice (spine in transparent purple, head in transparent yellow and PSD in green). (**G**) The PSD areas were plotted against the spine head volumes. A statistically significant positive correlation between the PSD area and the spine head volume was found in both genotypes (Spearman’s rank-order test: the *Phldb2*^+/+^ mice, n = 30 synapses, r = 0.879, *P* < 0.001; the *Phldb2*^−/−^ mice, n = 30 synapses r = 0.835, *P* < 0.001). The average PSD areas (**H**) and head volumes (**I**) were not significantly different between the *Phldb2*^−/−^ mice and the *Phldb2*^+/+^ mice [Spearman’s rank-order test, *P* = 0.598 for (**H**) and *P* = 0.330 for (**I**)].

PSD-95 is a scaffold protein that is found at the postsynaptic membrane specialization of the excitatory synapse and helps NMDA receptors localize at the postsynaptic membrane^23^ and BDNF is crucial for NMDA function^11–13^. Thus, we asked whether the localization of NMDA receptors at the postsynaptic membrane is altered in the *Phldb2*^−/−^ mice. We took advantage of the freeze-fracture replica immunolabelling (FRIL) technique for high-sensitivity quantitative detection of endogenous NMDA receptors at the surface of the dendritic spine with high spatial resolution. The postsynaptic membrane area in the plasma membrane of a dendritic spine was identified in the exoplasmic face of the replicas as an area accompanied by clustered intramembrane particles (IMP)^24^ labelled for the NR1 subunit of NMDA receptor (Fig. 3A,B). For both *Phldb2*^+/+^ and *Phldb2*^−/−^ mice, the number of immunogold particles for NR1 in individual IMP cluster areas was proportional to the area of the IMP clusters (r = 0.763, p < 0.001 for *Phldb2*^+/+^ mice and r = 0.680, p < 0.001 for *Phldb2*^−/−^ mice) (Fig. 3C). In contrast, significant reductions in the labelling density for synaptic NR1 were observed in the *Phldb2*^−/−^ mice (597 ± 21.7 gold particles/µm^2^ for *Phldb2*^+/+^ mice and 468 ± 22.0 gold particles/µm^2^ for *Phldb2*^−/−^ mice, Student’s *t*-test, *p* < 0.001) (Fig. 3D). Next, we reconstructed spines from serial electron micrographs captured by a focused ion beam scanning electron microscope (FIB-SEM) to investigate whether the decrease in labelling density for NR1 is due to an enlargement of PSD areas in the mutant mice. PSDs were observed as electron-dense thickenings of the postsynaptic plasma membrane, which is similar to their appearance in conventional transmission electron microscopy. The PSD region was traced with a green line in individual images, and the entire area of the postsynaptic membrane specialization as well as a spine head (yellow) was reconstructed (Fig. 3E,F, respectively). The area of the PSD was proportional to the volume of the spine head in both genotypes (r = 0.879, p < 0.001 for *Phldb2*^+/+^ mice and r = 0.835, p < 0.001 for *Phldb2*^−/−^ mice) (Fig. 3G). The average PSD areas (0.1 ± 0.009 µm^2^ for *Phldb2*^+/+^ mice and 0.09 ± 0.01 µm^2^ for *Phldb2*^−/−^ mice, Spearman’s rank-order test, *p* = 0.598) and spine head volumes (0.08 ± 0.013 µm^3^ for *Phldb2*^+/+^ mice and 0.09 ± 0.017 µm^3^ for *Phldb2*^−/−^ mice, Spearman’s rank-order test, *p* = 0.33) were not significantly different between the two genotypes (Fig. 3H,I). In Fig. 3H, the average PSD areas were not significantly different in the presence/absence of Phldb2, suggesting that Phldb2 does not have apparent effects on PSD.

### Phldb2 is essential for interaction between the NMDA receptor and CaMKII

Together, the NMDA receptor and CaMKII are crucial for LTP^5,6^, and the formation of a complex between the two is important for stable maintenance of LTP^25^. To our surprise, binding between CaMKIIα and NMDA receptors (NR2A, NR2B and NR1) was very weak in the absence of Phldb2 (Supplementary Fig. 6). This suggests that Phldb2 plays a crucial role in the interaction between CaMKIIα and the NMDA receptor, which is demonstrated to be essential for LTP induction^26^.

### Phldb2 contributes to GluA2 accumulation at the membrane surface of the spine through PIP_3_

**Figure 4:**
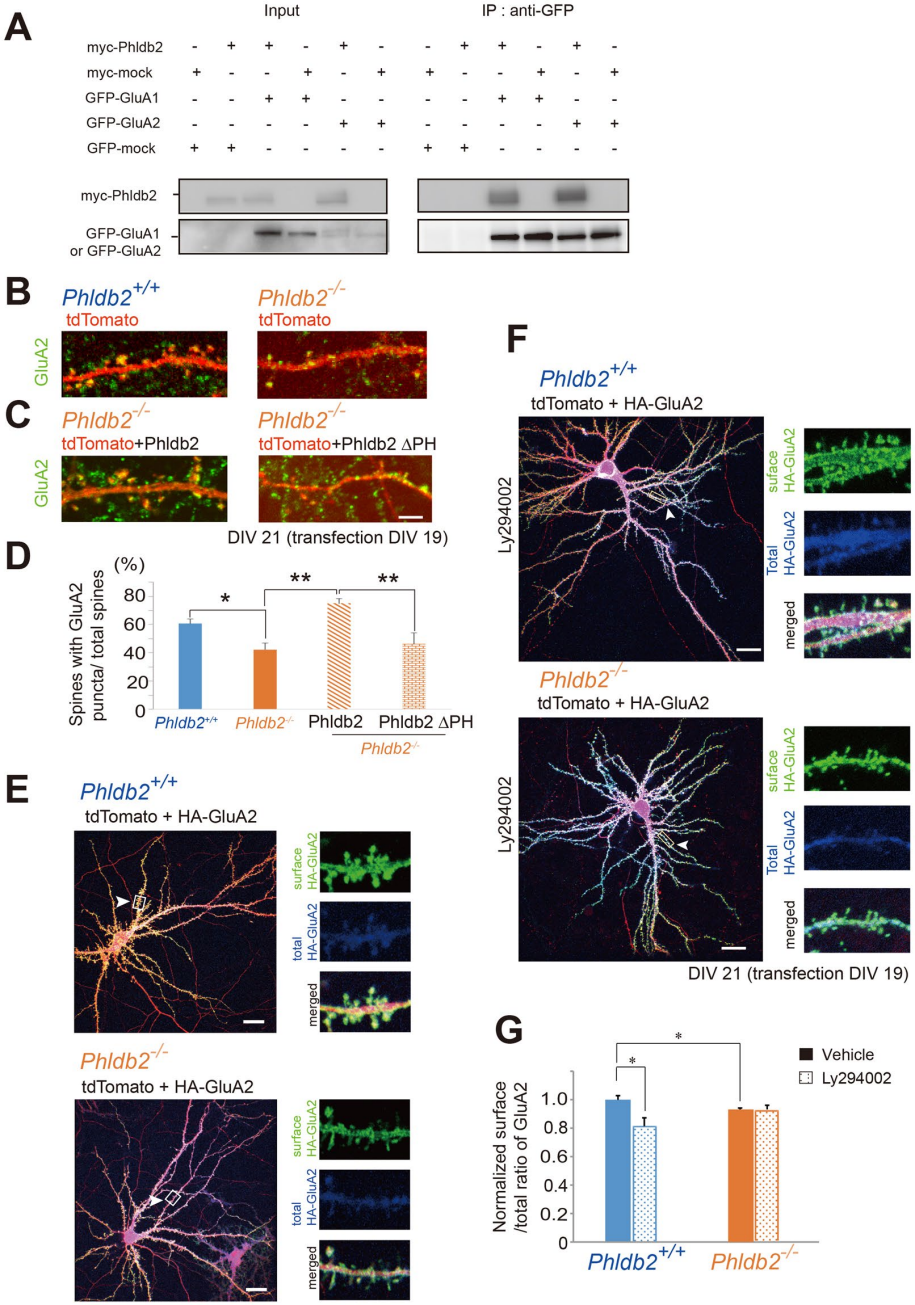
Phldb2 deletion results in a decrease in the number of GluA2-positive spines and the surface accumulation of GluA2. (**A**) Expression vectors of myc-Phldb2 or myc-mock, and GFP-GluA1 or GFP-GluA2 were co-transfected in COS-7 cells. Lysates were immunoprecipitated with anti-myc antibody. Phldb2 was co-immunoprecipitated with GluA1 and GluA2, and an amount of Phldb2 was increased in the presence of GluA1 or GluA2. The grouping of blots was cropped from Supplementary Fig. 7. (**B**) At DIV 21, endogenous GluA2 subunits were sparsely distributed in the spines of the *Phldb2*^−/−^ mouse hippocampal neurons. (**C**) For rescue experiments, hippocampal neurons of the *Phldb2*^−/−^ mice were transfected with tdTomato expression vectors and myc-Phldb2 or myc-Phldb2 ΔPH expression vector. Neurons were fixed at DIV 21. Endogenous GluA2 subunits were stained (green). (**D**) The number of spine-associated GluA2 puncta was significantly reduced in the *Phldb2*^−/−^ mice and was rescued by Phldb2 expression. (*Phldb2*^+/+^ mice, n = 348 spines; *Phldb2*^−/−^ mice, n = 187 spines; *Phldb2*^−/−^ mice with exogenous Phldb2, n = 283 spines; *Phldb2*^−/−^ mice with ΔPhldb2, n = 154 spines, Tukey-Kramer test, *P < 0.05, **P < 0.01). (**E**) Cultured hippocampal neurons were transfected with HA-GluA2 and tdTomato expression vectors at DIV 19. At DIV 21, HA-GluA2 at the membrane surface was visualized by staining for HA without Triton X-100 treatment (green). The neurons were then treated with Triton X-100, and total HA-GluA2 was observed (blue). Magnified images of the dendritic regions in white squares are shown in the right panels (arrowheads). (**F**) For Ly294002 treatment, 10 μM Ly294002 was added to the medium 60 min in advance of observation. (**G**) The fluorescence intensity of the surface GluA2 was divided by that of total GluA2 (surface/total ratio of GluA2) in the *Phldb2*^+/+^ neurons and was defined as 1.0 for normalization. The normalized surface/total GluA2 ratio was lower in the *Phldb2*^−/−^ mice than in the *Phldb2*^+/+^ mice. The surface/total ratio of GluA2 in the *Phldb2*^+/+^ mice was decreased by Ly294002 treatment, whereas the same was not true in the *Phldb2*^−/−^ mice (Mean ± SEM. n = 10 neurons for each group, Student’s *t*-test, *P < 0.05). Scale bars = 10 µm (**B**,**C**) and 20 µm (**E**,**F**).

It has been demonstrated that PIP_3_ controls synaptic function by maintaining AMPA receptor clustering at the postsynaptic membrane and is important for plasticity^9^, although the responsible molecules remain elusive. Our observation that Phldb2 bound to GluA1 or GluA2 (Fig. 4A and Supplementary Fig. 7) led us to ask whether Phldb2 is involved in this process.

In hippocampal neurons, GluA2 accumulated abundantly in the dendritic spines in the presence of Phldb2 (Fig. 4B,D and Supplementary Fig. 8A) (60.58% ± 3.23% for *Phldb2*^+/+^ mice and 42.27% ± 4.59% for *Phldb2*^−/−^ mice, Tukey-Kramer test, *p* = 0.036). We carried out rescue experiments by expressing Phldb2 or Phldb2ΔPH in primary culture neurons, taken from *Phldb2*^−/−^ mice. Endogenous GluA2 accumulation was rescued by Phldb2 expression in the *Phldb2*^−/−^ mice, whereas it accumulated less abundantly in the dendritic spines in the presence of Phldb2∆PH in the *Phldb2*^−/−^ mice (75.01% ± 3.22% for Phldb2 expression and 46.36% ± 7.91% for Phldb2∆PH expression, Tukey-Kramer test, *p* = 0.0021 as compared with Phldb2∆PH expression, and 0.0002 as compared with the *Phldb2*^−/−^ mice). (Fig. 4C,D and Supplementary Fig. 8), it is suggested that the PH domain of Phldb2 is crucial for localization of GluA2 in the spine. Furthermore, surface GluA2 was less abundant in the *Phldb2*^−/−^ mice than in the *Phldb2*^+/+^ mice (Fig. 4E,G) (1.00 ± 0.03 for *Phldb2*^+/+^ mice and 0.93 ± 0.01 for *Phldb2*^−/−^ mice, Student’s *t*-test, *p* = 0.043). The surface GluA2 was also decreased in neurons expressing Phldb2∆PH (Supplementary Fig. 9A–C). Indeed, we found that the surface GluA2 was significantly decreased in the *Phldb2*^+/+^ mice by Ly294002 treatment (Fig. 4E–G) (1.00 ± 0.03 for *Phldb2*^+/+^ mice and 0.81 ± 0.06 for *Phldb2*^+/+^ mice with Ly294002, Student’s *t*-test, *p* = 0.009). Moreover, Ly294002 did not induce any changes in the surface expression of GluA2 in the *Phldb2*^−/−^ mice (Fig. 4F,G) (0.93 ± 0.01 for *Phldb2*^−/−^ mice and 0.93 ± 0.03 for *Phldb2*^−/−^ mice with Ly294002, Student’s *t*-test, *p* = 0.96). Therefore, it is likely that Phldb2 works in the downstream of PIP_3_ signalling cascades as a mediator of the membrane surface expression of the AMPA receptor.

### Synaptic expression of the AMPA receptor is impaired in the *Phldb2*^−/−^ mice

**Figure 5:**
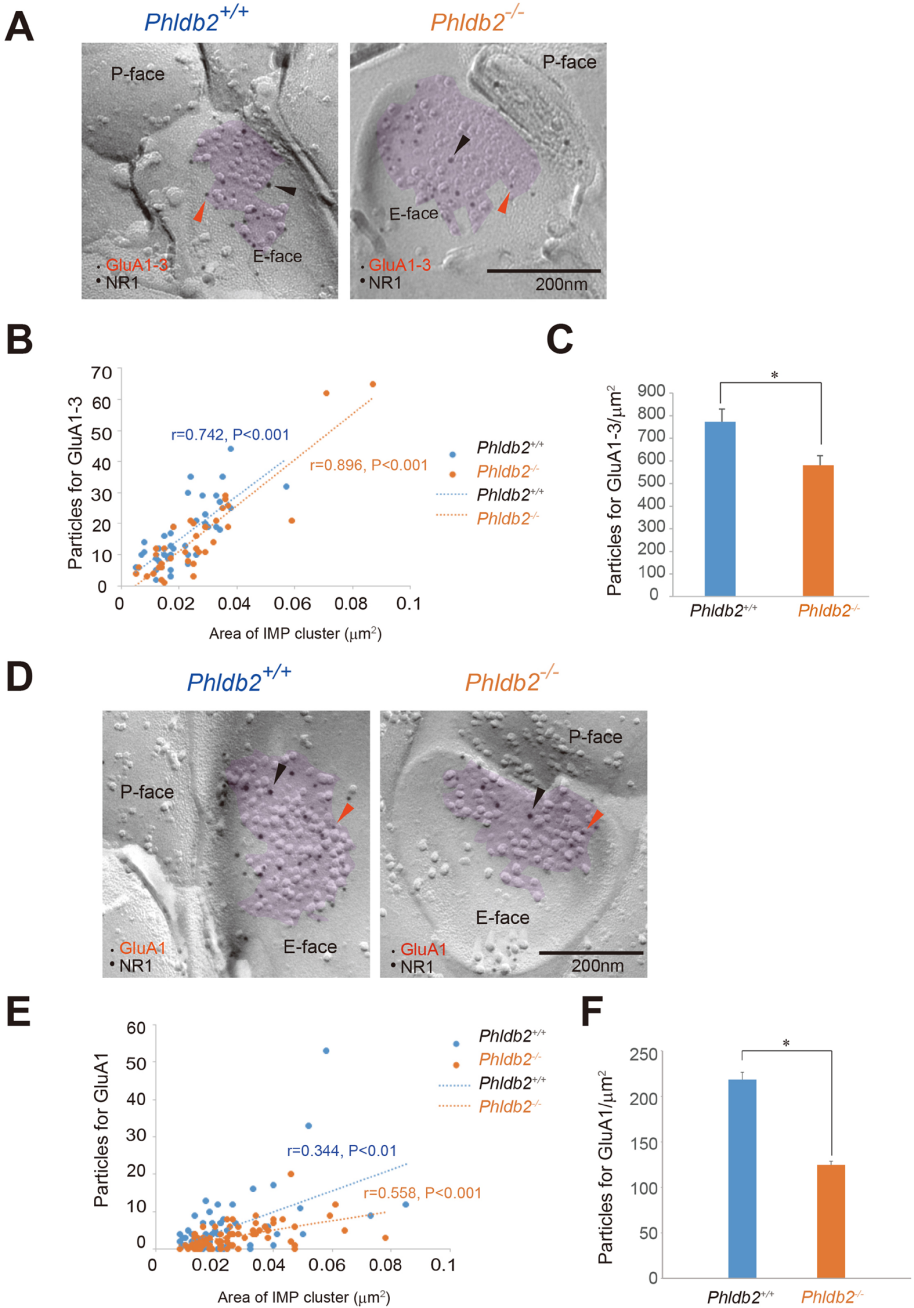
Phldb2 gene deletion results in a significant decrease in synaptic AMPA receptor density. Replicas were prepared from the CA1 region of the hippocampus and labelled for AMPA receptors ((**A**–**C**): GluA1–3; (**D**–**F**): GluA1) in combination with the NR1 subunit of NMDA receptors as a marker for excitatory synapses. The synaptic identity of these IMP cluster areas (purple area) was further confirmed by immunolabelling for the NR1 subunit visualized with 10 nm immunogold (black arrowheads in **A** and **D**). Immunoreactivity for GluA1–3 or GluA1 was visualized with 5-nm immunogold particles (orange arrowheads) in (**A**,**D**), respectively. (**B**,**E**) The numbers of immunoparticles for GluA1–3 (**B**) or GluA1 (**E**) in individual IMP clusters were plotted against the IMP cluster areas. In both cases, a statistically significant positive correlation between the AMPAR labelling numbers and synaptic areas was found regardless of genotype (Pearson’s correlation test for GluA1–3: the *Phldb2*^+/+^ mice, n = 43 synapses, r = 0.742, *P* < 0.001; the *Phldb2*^−/−^ mice, n = 36 synapses, r = 0.896, *P* < 0.001, and Spearman’s rank-order test for GluA1: the *Phldb2*^+/+^ mice, n = 69 synapses, r = 0.344, *P* < 0.01; the *Phldb2*^−/−^ mice, n = 69 synapses, r = 0.558, *P* < 0.001). However, significant reductions in synaptic GluA1–3 (**C**) and GluA1 (**F**) labelling densities were detected in the *Phldb2*^−/−^ mice compared with the *Phldb2*^+/+^ mice (Mean ± SEM. Student’s *t*-test for GluA1–3, *P < 0.05, Spearman’s rank-order test for GluA1, *P < 0.05).

To directly evaluate whether lack of Phldb2 affects AMPA receptor expression at the postsynaptic membrane, we took advantage of the FRIL technique to monitor endogenous AMPA receptors at the surfaces of the spines with high spatial resolution. We examined the subcellular localization of AMPARs (GluA1–3)^27^ in the stratum radiatum of mouse hippocampal CA1 with or without subunit selectivity by anti-GluA1 or anti-GluA1–3 antibodies, respectively. The postsynaptic membrane area was identified in the exoplasmic face of replicas, accompanied by clustering of IMP labelled for NR1 subunit of NMDA receptors (Fig. 5A,C). For both genotypes, the number of immunogold particles for GluA1–3 (r = 0.742, *P* < 0.001 for *Phldb2*^+/+^ mice and r = 0.896, *P* < 0.001 for *Phldb2*^−/−^ mice) (Fig. 5B) and GluA1 (r = 0.344, *P* < 0.01 for *Phldb2*^+/+^ mice and r = 0.558, *P* < 0.001 for *Phldb2*^−/−^ mice) (Fig. 5E) in the IMP cluster areas of each synapse was proportional to the size of the IMP clusters. In contrast, significant reductions in the labelling density for synaptic GluA1–3 (Fig. 5C) (773 ± 56.7 gold particles/µm^2^ for *Phldb2*^+/+^ mice and 581 ± 42.3 gold particles/µm^2^ for *Phldb2*^−/−^ mice, Student’s *t*-test, *p* = 0.012) and GluA1 (Fig. 5F) (218 ± 8.2 gold particles/µm^2^ for *Phldb2*^+/+^ mice and 125 ± 4.2 gold particles/µm^2^ for *Phldb2*^−/−^ mice, Spearman’s rank-order test, *p* = 0.025) were observed in the *Phldb2*^−/−^ mice, confirming that AMPAR trafficking mechanisms responsible for rapid changes in synaptic strength were impaired by the lack of Phldb2. It is noteworthy that the reduction of labelling density was more evident in the GluA1 analysis than in the GluA1–3 analysis, suggesting that GluA1-containing AMPARs were remarkably affected by the Phldb2 deficiency.

### Phldb2 is necessary for LTP induction in the hippocampus

**Figure 6:**
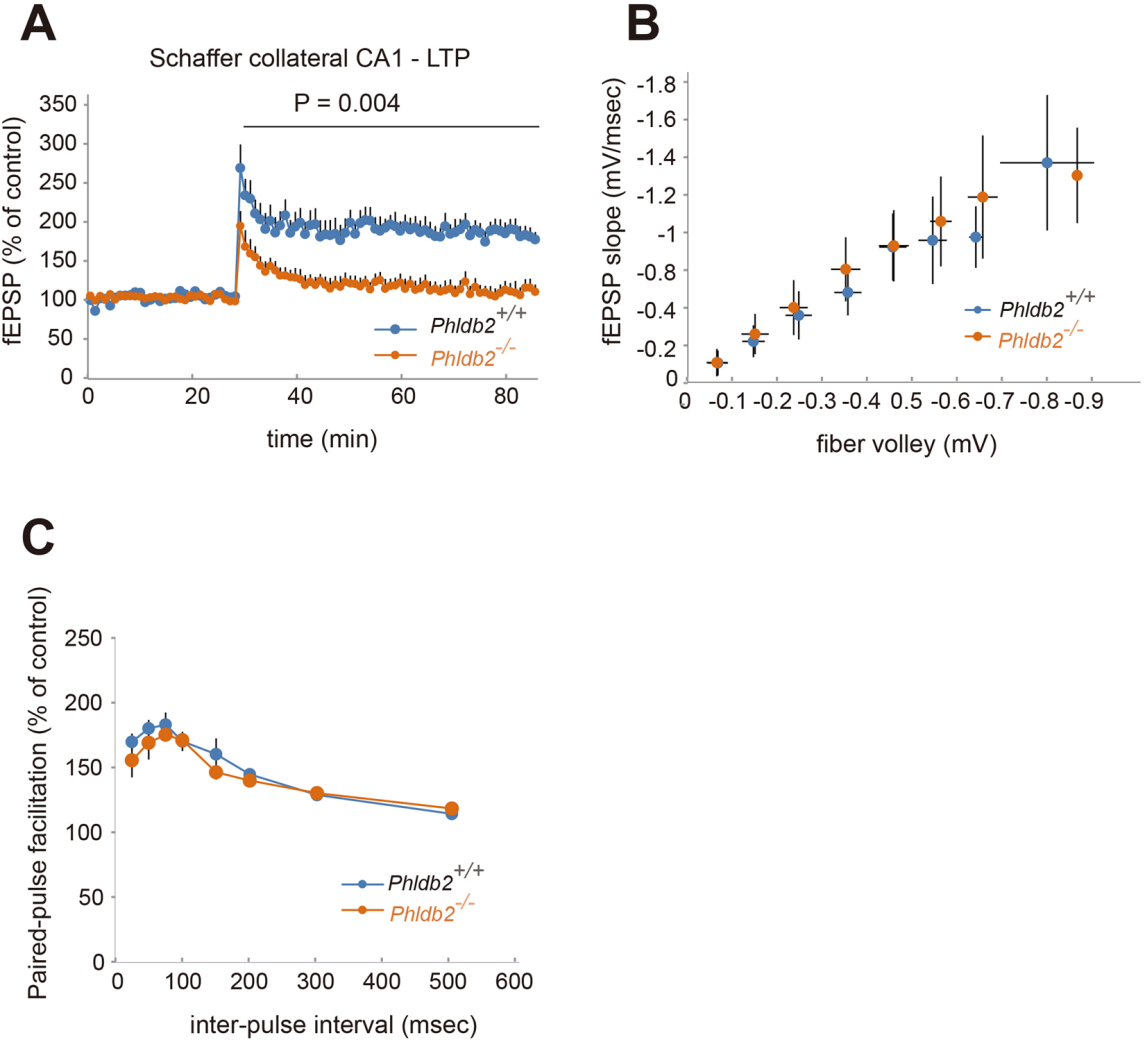
Phldb2 is necessary for LTP induction. (**A**) Electrophysiological analyses on the effects of Phldb2 on hippocampal LTP; time course of the normalized fEPSP slope recorded in slices from the *Phldb2*^+/+^ mice and the *Phldb2*^−/−^ mice. LTP was induced by high-frequency stimulation (HFS) (100 Hz; 100 pulses, 1 sec) of the Schaffer collaterals. LTP was induced in the *Phldb2*^+/+^ slices (n = 8) but not the in *Phldb2*^−/−^ slices (n = 6) (Mean ± SEM. Two-way repeated measures ANOVA, F _(1,12)_ = 1.09, *P* = 0.0035). One hundred percent corresponds to the pre-LFS baseline. (**B**) The input-output curve of fEPSP slope (mV/ms) versus presynaptic fibre volleys (FV; mV) at the Schaffer collateral pathway did not differ between *Phldb2*^+/+^ slices (n = 10) and *Phldb2*^−/−^ slices (n = 13) (Mean ± SEM. One-way ANOVA analysis, F _(1,225)_ = 0.7596, *P* = 0.3844). (**C**) Paired-pulse facilitation, the short-term enhancement of synaptic efficacy following the delivery of two closely spaced stimuli, did not significantly differ between *Phldb2*^+/+^ slices (n = 10) and *Phldb2*^−/−^ slices (n = 13) (Mean ± SEM. One-way ANOVA analysis, F _(1,171)_ = 1.431, *P* = 0.233).

Since the behaviour of synaptic AMPA receptor is essential for synaptic plasticity and that PIP_3_ is demonstrated to be crucial for LTP induction^9,10^, we asked whether Phldb2 is involved in LTP induction. LTP was induced in the CA1 neurons of hippocampal slices by high-frequency stimulation (HFS) of Schaffer collaterals. The prolonged elevation of recorded field excitatory postsynaptic potentials (fEPSPs) was abolished in the *Phldb2*^−/−^ mice (Fig. 6A) (two-way repeated measures ANOVA, *p* = 0.004), suggesting that Phldb2 is required for LTP induction. In addition, to ascertain whether Phldb2 is solely involved in postsynaptic terminals, we used extracellularly recorded fEPSPs and paired-pulse facilitation (PPF) to assess the strength of basal synaptic transmission and the presynaptically mediated form of potentiation, respectively. The strength of this connection was quantified for each slice by measuring the fEPSP slope and dividing this value by the corresponding fibre volley amplitude at each input stimulation level. No significant difference was observed in terms of basal synaptic function between the *Phldb2*^+/+^ mice and the *Phldb2*^−/−^ mice (Fig. 6B,C). Therefore, it is likely that Phldb2 works in postsynaptic terminals. Densities of some synaptic glutamate receptors decreased in the *Phldb2*^−/−^ mice (Figs 3–5). It is difficult to assess how much such decreases affected, but it is possible that the subunit composition of extrasynaptic surface pools of AMPARs may be aberrant in the absence of Phldb2, just like PICK1 KO mice^28,29^.

### Reference memory is impaired in the *Phldb2*^−/−^ mice

**Figure 7:**
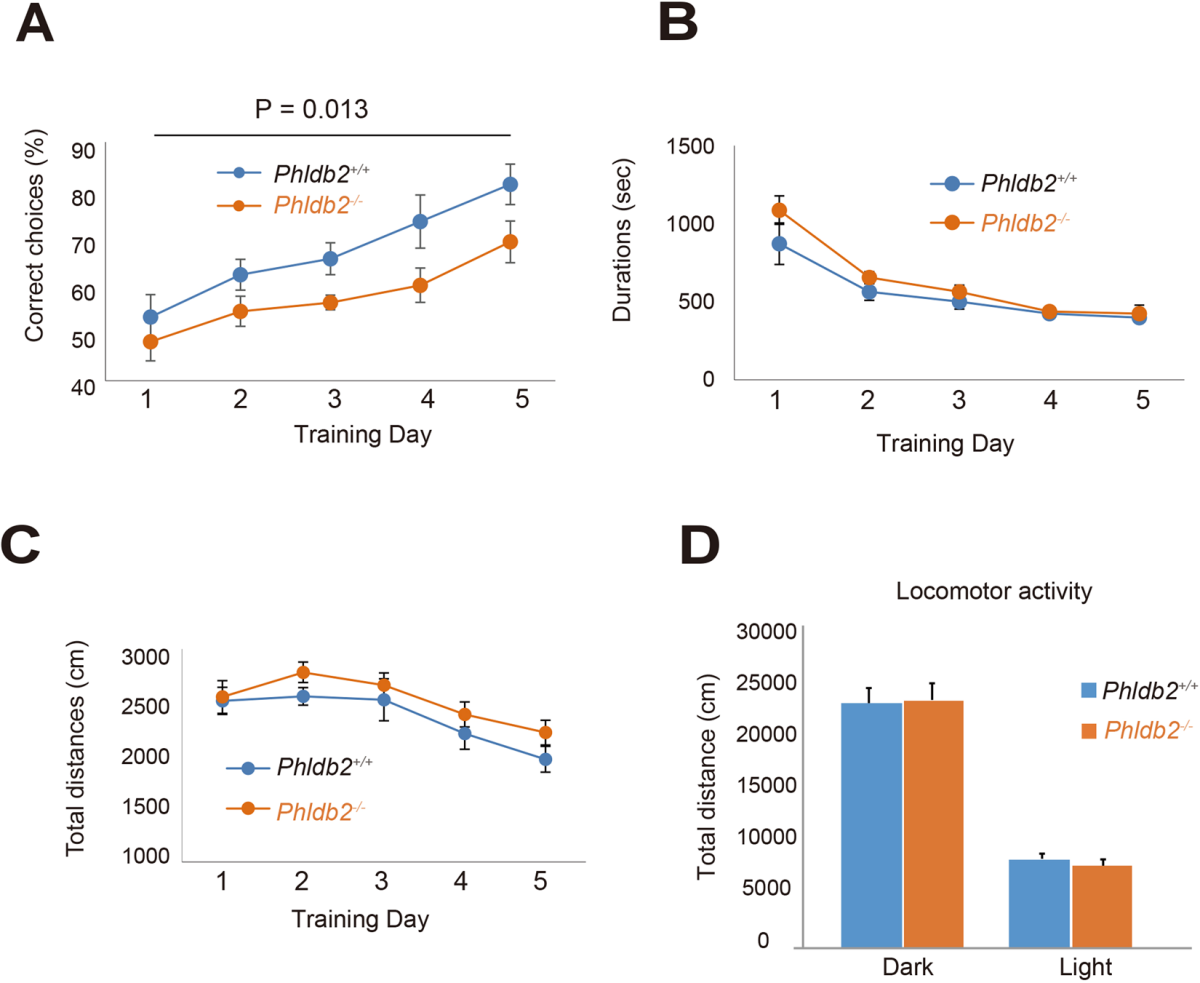
Deletion of Phldb2 impairs performance on the T-maze left-right discrimination test. (**A**–**C**) Left-right discrimination in a T-maze test was examined. The graph shows the percentage of correct choices (**A**), duration (**B**) and total distance (**C**). The *Phldb2*^−/−^ mice (n = 11) made significantly fewer correct choices than the *Phldb2*^+/+^ mice (n = 9). (Mean ± SEM. Two-way repeated measures ANOVA, F _(1,18)_ = 7.69, *P* = 0.013). The average duration and total distance were not significantly different between the *Phldb2*^−/−^ mice and *Phldb2*^+/+^ mice [Mean ± SEM. Two-way repeated measures ANOVA, F _(1,17)_ = 1.625, *P* = 0.220 for (**A**) and F_(1,17)_ = 1.614, *P* = 0.221 for (**B**)]. (**D**) No obvious differences were observed in the locomotor activity of the two genotypes. The total distance moved in the 24-hr locomotor test did not differ between the *Phldb2*^−/−^ mice and the *Phldb2*^+/+^ mice (Mean ± SEM. Student’s *t*-test, n = 14 for each mice, *P* = 0.660 for dark and *P* = 0.330 for light).

T-maze is well established as a behavioral task sensitive to hippocampal lesions and to subtle manipulations of hippocampal synaptic plasticity^30^. We examined the *in vivo* roles of Phldb2 in mice. A automatic T-maze left-right discrimination task (Fig. 7A–C) and 24-hr home cage monitoring (Fig. 7D) were implemented. For reference memory, both the *Phldb2*^+/+^ mice and the *Phldb2*^−/−^ mice gradually improved their performance over the course of the training period, but the *Phldb2*^−/−^ mice made significantly fewer correct choices than the *Phldb2*^+/+^ mice (Fig. 7A) (two-way repeated measures ANOVA, *p* = 0.013), suggesting that reference memory was impaired. On the other hand, duration time and total distances were not significantly different between the *Phldb2*^+/+^ mice and the *Phldb2*^−/−^ mice, suggesting that the locomotor activity and movement to the task were normal even in the *Phldb2*^−/−^ mice. Furthermore, in terms of locomotor activity in the home cage, we did not find any significant differences in the *Phldb2*^−/−^ mice (Fig. 7D).

## Discussion

It has been demonstrated that BDNF acts on the dendritic spines through trkB and is crucial for LTP^11–13,21,31^. Since PI3K works in the downstream signalling cascades of the trkB receptor^32^ and Phldb2 moved into the spines in response to PIP_3_, it is likely that Phldb2 mediates PI3K activity downstream of the trkB receptor, in addition to AMPA receptor-associated PI3K^33^, in the process of LTP.

**Figure 8:**
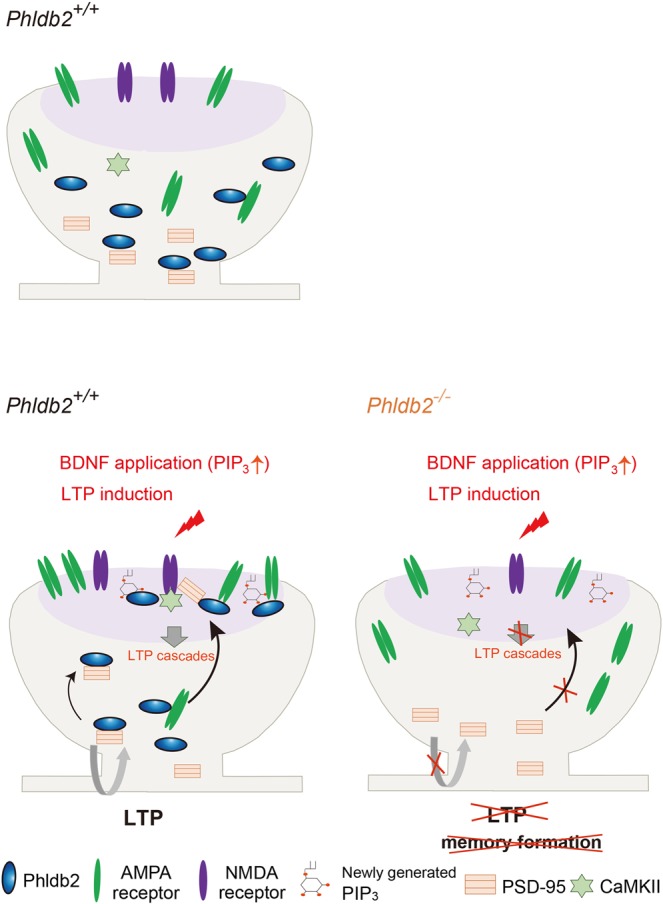
Summary and possible roles of Phldb2 for synaptic plasticity in the spine. Postsynaptic membrane area is shown in purple.

Transient elevation of PIP_3_ levels on the postsynaptic membrane during LTP is elegantly measured^10,34^. The identification of Phldb2 as a PIP_3_-sensing molecule in the dendritic spine enables us to understand how glutamate receptors and postsynaptic events are regulated for synaptic plasticity (Fig. 8). It has been shown that calcium influx through NMDA receptors leads to CaMKII activation at the induction phase of LTP and that CaMKII is kept active by binding to NMDA receptor subunits (NR2B, NR1), regardless of its phosphorylation status^35^. In the present study, we observed the essential role of Phldb2 in binding between NMDA receptors and CaMKII.

Our observation clearly shows the significance of Phldb2 for synaptic plasticity. In addition, the reduction in postsynaptic membrane-expressed NR1 in the *Phldb2*^−/−^ mice with no substantial changes in spine volume or PSD areas further supported the importance of Phldb2 for LTP induction.

PSD-95 is a scaffolding protein that is highly stable at the synapse^36,37^. PSD-95 binds to NMDA receptors and stabilizes NMDA receptors in post synapse, which is necessary for the induction of LTP^5^. PIP_3_ regulates the localization of PSD-95 in the dendritic spine^9^. We showed here that Phldb2 helps PSD-95 localize in the spine head, therefore it is likely that PIP_3_ regulates the behaviour of PSD-95 via Phldb2. We observed that the postsynaptic membrane-expressed NR1 was reduced in the *Phldb2*^−/−^ mice. Taken these things into consideration, Phldb2 is likely to regulate NMDA receptor stabilization in the postsynaptic membrane as well as its trafficking to synapse through PSD-95.

In addition, PSD-95 deletion enhances LTP^38^, whereas PSD-95 overexpression occludes LTP^39^. Appropriate levels of PSD-95 are required for activity-dependent synapse stabilization after initial phases of synaptic potentiation^40^. We demonstrated that turnover (mobility and/or degradation) of PSD-95 was changed in the *Phldb2*^−/−^ mice, suggesting that Phldb2, together with PIP_3_, is important for this event.

In the subsequent maintenance phase of LTP, PI3Ks including AMPA receptor-associated PI3K are demonstrated to be crucial for long-lasting facilitation of AMPA receptor insertion into the membrane^9,10^, but which molecules work together with PIP_3_ and the nature of their interactions had not been identified. We observed that facilitation of AMPA receptor insertion was impaired in the absence of Phldb2 (or in the presence of Phldb2 without its PH domain). It is demonstrated that PSD-95 influences synaptic AMPA receptor content^41,42^. Therefore, it is likely that Phldb2 regulates the behaviour of AMPA receptors via PSD-95 during LTP. Furthermore, it has been shown that AMPA receptor-associated PI3K plays a role in the enhancement of LTP and that PI3K activity is required for AMPA receptor surface expression^33^. Thus, it is possible that Phldb2 is also crucial in this process. Since the *Phldb2*^−/−^ mice exhibited impaired memory, it is likely that the late phase of LTP, in which protein synthesis is crucial, was also damaged in the absence of Phldb2. Therefore, Phldb2 plays an essential role in every phase of LTP, and the identification of Phldb2 allows us to understand how PIP_3_ is involved in synaptic plasticity.

A role of Phldb2, or LL5β, in the neuro-muscular junction is reported^43,44^. In the neuro-muscular junction, Phldb2 is concentrated at synaptic sites, associated with the postsynaptic membrane and results in the high acetylcholine receptor aggregation. Moreover, it is shown that the high density of acetylcholine receptor in the postsynaptic muscle membrane is regulated by agrin through PI3 kinase, GSK3β, CLASP2 and Phldb2^44^. Here, we first showed that Phldb2 plays a role in the trafficking of synaptic proteins in the central nervous system, especially for glutamate receptors. Together with their data and our data, it is probable that Phldb2 regulates the receptor accumulation in the postsynaptic membrane via PIP_3_ throughout the nervous system, in the central as well as the peripheral nervous system.

In conclusion, the versatile protein Phldb2 is a critical mediator of synaptic plasticity, sensing membrane phosphoinositides (Fig. 8).

## Materials and Methods

### Animals

The Phldb2 mutant was established (Accession No. CDB0791K: http://www2.clst.riken.jp/arg/mutant%20mice%20list.html). To inactivate Phldb2 in the germ line, we crossed the mice carrying a floxed allele with TNAP-Cre knock-in mice. C57BL/6 mice (SLC, Hamamatsu, Japan) and Phldb2 knockout mice were housed at a constant temperature and humidity and were provided with food and water *ad libitum*. Male mice were used. All experimental procedures were approved by Animal Research Committee, University of Fukui, the Institutional Animal Care and Use Committee of Nara Institute of Science and Technology, and the Institutional Animal Care and Use Committee of Maebashi Institute of Technology. All experiments were conducted in compliance with the institutional guidelines and regulations of them. All efforts were made to minimize both the number of animals used and their suffering.

### Cell culture

Primary hippocampal neurons were prepared from E17 embryos as previously described^45^.

### Photoactivation

Photoactivation experiments were performed with PAGFP-PSD-95 at the synapses^33^.

### SDS-digested freeze-fracture replica immunolabelling (FRIL)

Brain slices (130 μm) were prepared from the hippocampi of post-natal day 56 mice for FRIL. Mice were perfused transcardially for 1 min with PBS, followed by 12 min of perfusion with 0.1 M PB containing 2% paraformaldehyde and 15% saturated picric acid solution at a rate of 5 ml/min. The brains were quickly removed from the skull and sliced (130 µm thick) on a vibratome (Dosaka, Kyoto, Japan). Hippocampal slices were cryoprotected in 30% glycerol in 0.1 M PB and high-pressure frozen using HPM010 machine (Bal-Tec, Balzers, Liechtenstein). The frozen slices were then freeze fractured at −130 °C and replicated with an initial carbon layer (5 nm), shadowed unidirectionally with platinum (2 nm), and strengthened with a second carbon layer (15 nm) in a BAF060 freeze-etching machine (Bal-Tec). After thawing, the tissue attached to the replicas was solubilized by shaking at 80 °C for 18 hr in the following solubilisation solution: 15 mM Tris[hydroxymethyl]-aminomethane, 20% sucrose, and 2.5% sodium dodecyl sulfate, pH 8.3. Immunolabelling of replicas was carried out according to previously published procedures with minor modifications^46^. Blocking was performed with a solution consisting of 5% bovine serum albumin and 0.1% TWEEN 20 in TBS (pH 7.4). The replicas were incubated in primary antibodies (anti-GluA1–3 or anti-GluA1 antibodies, both generated in horse against synthetic peptides deduced from the common and unique aa sequences of the extracellular portion of GluA1, respectively) at 15 °C for 3 days. The specificity of these antibodies in FRIL analysis was confirmed by the absence of labelling in parallel fibre-Purkinje cell synapses of GluA2/3 knock-out mice and hippocampal synapses of GluA1 knock-out mice. Following extensive washing with unbound primary antibody, the replicas were incubated with gold-conjugated anti-rabbit secondary antibodies (British Biocell International, Cardiff, UK; 5 nm), overnight at 15 °C. To mark IMP clusters on exoplasmic-face derived from excitatory synapses, NMDA receptor labelling was carried out simultaneously with the secondary antibody incubation by adding mouse anti-NR1 antibody (clone 54.1, 1:100, Millipore), which was then detected by incubation with anti-mouse secondary antibodies (British Biocell International; 10 nm) at room temperature for 1 hr. The replicas were then mounted on pioloform-coated copper mesh grids and examined at 80 kV acceleration voltage in an H-7650 transmission electron microscope equipped with a CCD camera (Hitachi High-Technologies Corporation, Tokyo, Japan). Electron micrographs captured at 40,000x were analysed with the program ImageJ (Rasband, W.S., ImageJ, U.S. National Institutes of Health, Bethesda, MA, http://imagej.nih.gov/ij/, 1997–2015) for measurement of synaptic area and quantification of immunogold particles within individual synapses. The numbers of synapse analysed in this study are as follows: 62 synapses (NR1 of the *Phldb2*^+/+^ mice and the *Phldb2*^−/−^ mice), 43 synapses (GluA1–3 of the *Phldb2*^+/+^ mice), 36 synapses (GluA1–3 of the *Phldb2*^−/−^ mice), and 69 synapses (GluA1 of the *Phldb2*^+/+^ mice and the *Phldb2*^−/−^ mice).

### Focused ion beam scanning electron microscopy (FIB-SEM)

Mouse brains for FIB-SEM observation were prepared as previously described^47^ with some modifications. Mice were perfused with 0.1 M phosphate buffer (PB, pH 7.4) containing 1.5% glutaraldehyde and 0.8% paraformaldehyde at a rate of 5 ml/min for 12 min, and then the brains were dissected and postfixed in 4% paraformaldehyde in 0.1 M PB overnight at 4 °C. Coronal brain slices (100 µm thick) were prepared with a vibratome (Dosaka), and those containing the dorsal hippocampus were fixed in an aqueous solution of 2% osmium tetraoxide/1.5% potassium ferrocyanide in aqueous solution on ice for 1 hr. Sections were treated with thiocarbohydrazide solution for 20 min at room temperature and then fixed in aqueous osmium tetraoxide (2%) for 30 min at room temperature followed by 1% uranyl acetate overnight at 4 °C. For en bloc lead staining, the sections were placed in Walton’s lead aspartate solution for 75 min at 60 °C. The sections were dehydrated in a graded series of ethanol solutions (50%, 70%, 90%, 90%, and 100% for 5 min each), followed by propylene oxide 2 times for 10 min, and then Durcupan ACM resin (Fluka Durcupan ACM kit, Sigma-Aldrich) was infiltrated into the tissue by sequential treatment with 25%, 50%, and 75% Durcupan-propylene oxide mixture (2 hr each) and 100% Durcupan overnight at room temperature. The sections were transferred into fresh 100% Durcupan, flat embedded on a slide glass and then polymerized at 60 °C for 2 days. A piece of resin containing the CA1 area of hippocampus was trimmed out and re-embedded into a resin block, and the tissue was exposed with an ultramicrotome (Leica). The resin block was glued to a metal rivet with superglue, and the surface of resin block was coated with a 5-nm-thick carbon layer using a freeze-etch machine (BAF060, BAL-TEC) for better electron conductivity. Dendritic spines in the stratum radiatum of the hippocampal CA1 area (100–200 μm from the pyramidal cell layer) were imaged at 17500x magnification with 15-nm z-steps using a focused ion beam SEM (Scios, FEI, Eindhoven, Netherlands). The spatial resolutions of the original image in the X, Y and Z axis are 1.93, 2.45 and 15 nm/pixel, respectively. Serial images were aligned using a registration macro in the Fiji distribution of ImageJ^48^.

### Ultrastructural reconstructions

Three-dimensional reconstruction of dendritic spines was carried out with the aid of reconstruct software^49^ (Reconstruct 1.1.0.0, available from https://synapseweb.clm.utexas.edu). Independent traces were drawn for the entire spine structure, spine head and postsynaptic density (PSD) of mushroom spines, and three-dimensional volumes and area were obtained. The narrowest part of a spine was identified as a neck of the spine, and the most distal portion of the spine from the neck was considered a spine head for volume measurement. Thirty randomly chosen synapses per genotype for the *Phldb2*^+/+^ mice and the *Phldb2*^−/−^ mice were analysed in this study.

### Electrophysiology and Input-output relationship and paired-pulse facilitation

Experiments were performed according to the methods described with some modifications^50^.

### Behavioural Tests

The 24-hr locomotor test and the left-right discrimination test were performed as described^51^.

### Experimental Design and Statistical Analysis

All mice were male, and experimental groups were age-matched. All statistical analyses were performed using IBM SPSS statistics standard 23 and JMP pro 14. Pairwise comparisons between groups were conducted using the two-tailed Student’s *t-*test or N pair test, and correlations were tested for statistical significance by Pearson’s correlation test or Spearman’s rank-order test. Behavioural data were assessed for statistical significance by two-way repeated measures ANOVA analysis of variance. The null hypothesis was rejected at *p* < 0.05. Quantitative data are presented as the mean ± SEM.

## Supplementary information

supplementary information

## Data Availability

The datasets generated and analysed during the current study are available from the corresponding authors upon reasonable request.
