# Supplementary material for: PIP3-Phldb2 is crucial for LTP regulating synaptic NMDA and AMPA receptor density and PSD95 turnover
Source: Sci Rep. 2019 Mar 13;9:4305. doi: 10.1038/s41598-019-40838-6 (PMC6416313; doi:10.1038/s41598-019-40838-6)
Supplement: Supplementary file 1 — supplementary information [file 41598_2019_40838_MOESM1_ESM.docx]

**PIP_3_-Phldb2 is crucial for LTP regulating synaptic NMDA and AMPA receptor density and PSD95 turnover**

Min-Jue Xie, Yasuyuki Ishikawa, Hideshi Yagi, Tokuichi Iguchi, Yuichiro Oka, Kazuki Kuroda, Keiko Iwata, Hiroshi Kiyonari, Shinji Matsuda, Hideo Matsuzaki, Michisuke Yuzaki, Yugo Fukazawa and Makoto Sato

**Supplementary information**

**Methods**

***In situ* hybridisation**

For *in situ* hybridization, cryostat sections (16 μm) of paraformaldehyde fixed adult mouse brain were fixed with 4% paraformaldehyde in PBS (pH 7.4) for 10 min at room temperature. Endogenous alkaline phosphatase (AP) was inactivated in 0.2 M HCl for 10 min. The sections were then digested with 8 μg/ml of proteinase K (Roche) in PBS for 10 min at 37°C, and treated with 5 mM acetanhydride in 0.1 M triethanolamine (pH 8.0) for 10 min. Between each step, slides were rinsed with PBS for 5 min at room temperature. Hybridization was performed overnight (16 hrs) at 55°C in 50% formamide, 5× standard saline citrate (SSC; 20× SSC is 3 M NaCl and 0.3 M sodium citrate, pH 7.0), and 0.2 mg/ml bakers’ yeast tRNA. Washes were as follows: 30 min 2× SSC at room temperature, 30 min with 2× SSC at 65°C, 30 min with 0.2× SSC at 65°C twice. Bound probe was detected with anti-Digoxigenin-AP Fab fragment (Roche) using nitroblue etrazolium chloride/5-bromo-4-chloro-3-indolyl-phosphate as colour substrate. The antisense probe was transcribed from the 500bp cDNA fragment (nucleotides 1–500), 500bp cDNA fragment (nucleotides 838-1337) and 822bp cDNA fragment (nucleotides 665-1487) of Phldb2.

**Cell culture, DNA transfection and immunoprecipitation**

Primary hippocampal neurons were prepared from E17 embryos as previously described^45^. COS-7 cells were maintained in Dulbecco’s modified Eagle’s medium (Invitrogen) containing 10% fetal bovine serum at 37°C in 5% CO_2_. Two micrograms per 6 cm dish of expression vectors were transfected with FuGENE 6 transfection reagent (Roche Diagnostics Corporation, Indianapolis, IN). Forty-eight hours after transfection, cells were lysed in RIPA buffer (0.1% sodium deoxycholate, 0.5% Nonidet P-40, 150 mM NaCl, 50 mM Tris-HCl with 1% protease inhibitor cocktail). Lysis was removed insoluble materials by ultracentrifugation (60 min centrifugation at 100,000 x g) prior to IP experiments. Co-immunoprecipitation was performed with Dynabeads protein G (Invitrogen). The samples were incubated with an anti-myc, anti-GFP (MBL Medical and Biological Laboratories, Nagoya, Japan) or anti-CaMKIIα antibody (6G9, Abcam, Cambridge, UK) for 2 hr at 4 °C. Then, the Dynabeads were washed three times in ice-cold RIPA buffer and boiled in SDS sample buffer. The resulting sample was then resolved by SDS-PAGE. Proteins were subjected to western blot analysis.

**Immunohistochemistry (IHC)**

Paraffin-embedded brain tissue sections (3 µm) were deparaffinised, and the slides were washed in phosphate-buffered saline (PBS). IHC staining was performed using the avidin-biotin complex (ABC) technique and DAB staining to detect of Phldb2. For fluorescent IHC, images were obtained on a laser scanning microscope (LSM) 5 PASCAL confocal microscope (Zeiss, Tokyo, Japan). The antibodies used were mouse anti-NeuN antibody (MAB377, Millipore, Billeraca, MA) and rabbit anti-Phldb2 antibody.

**Production of Phldb2 monoclonal and polyclonal antibodies**

A monoclonal antibody against Phldb2 was prepared by standard techniques using the splenocytes of mice (C57BL/6 hybrids) immunized with a fragment of recombinant mouse Phldb2 (amino acids 418–760) produced in bacteria. Myeloma was utilized as a fusion partner to generate hybridomas, using standard selection and single-cell cloning techniques. A polyclonal antibody against Phldb2 was prepared by standard techniques by Sigma - Aldrich (St. Louis, MO); the immunization used the same fragment of recombinant mouse Phldb2 used for monoclonal antibody production. The antibodies were initially characterized by ELISA and immunoblotting.

**Preparation of synaptic membrane fractions**

Synaptic membrane fractions were obtained as previously described[^52^](#_ENREF_1). Mouse hippocampal homogenates were prepared by homogenizing the hippocampi in a buffer containing 10 mM HEPES (pH 7.4), 0.32 M sucrose and protease inhibitor cocktail. The solution was layered over a sucrose gradient consisting of two discontinuous layers of 0.8 M and 1.2 M sucrose dissolved in 10 mM Tris, pH 7.4, and centrifuged at 100,000 x *g* (28,700 rpm) for 2 hr in a Beckman SW 55Ti. The crude synaptic membrane fraction at the 0.8 M-1.2 M sucrose interface was collected. For the synaptic membrane fraction, the crude synaptosome fraction was lysed in hypotonic solution consisting of 10 volumes of 10 mM HEPES, (pH 7.4) and protease inhibitor, and osmotic shock was carried out at 4 °C for 45 min under gentle stirring. The synaptic membrane fraction was obtained by centrifugation at 33,000 x *g* (16,500 rpm) for 20 min. For the purified synaptosome fraction, the crude synaptosome fraction was lysed in 2 volumes of a buffer containing 10 mM HEPES (pH 7.4), 0.32 M sucrose and protease inhibitor, then centrifuged at 33,000 x *g* (16,500 rpm) for 20 min.

**Generation of Phldb2 knockout mice**

The Phldb2 mutant (Accession No. CDB0791K: http://www2.clst.riken.jp/arg/mutant%20mice%20list.html) was established as follows: Diphtheria toxin fragment A (DT-A)/conditional KO FW (http://www.cdb.riken.jp/arg/cassette.html) was used for the construction of a targeting vector. A targeting vector for Phldb2 was electroporated into TT2 embryonic stem cells (ES cells). Correctly targeted ES cells (with a floxed allele) were identified by southern blot analysis or polymerase chain reaction (PCR) (data not shown). The floxed region contained exon 2. The ES cells were injected into blastocysts to generate germline chimaeras.

To inactivate Phldb2 in the germ line, we crossed the mice carrying a floxed allele with TNAP-Cre knock-in mice. The knockout mice were maintained on a C57BL/6J genetic background. The mice with the targeted allele were genotyped by PCR using the following primer pair: 5'-ccagagactgaaacactgggcg-3' and 5'-atcttctgcca tgcatgtggct-3'. We also confirmed the deletion of exon 2 of the *Phldb2* gene by PCR with the primers 5'-ccagagactgaaacactgggcg-3' and 5'-ggaaaagcgcctcccctacccg-3'.

**Immunostaining**

Primary neuronal cultures were fixed with 4% paraformaldehyde, and staining was performed as previously described^39^. Images were obtained on a LSM 5 PASCAL confocal microscope. The antibodies used were as follows: mouse anti-PSD-95 antibody, mouse anti-GluA2 antibody (MAB397, Millipore)^53,^[^54^](#_ENREF_3), rabbit anti-Phldb2 antibody, and mouse anti-myc antibody (sc-40, Santa Cruz Biotechnology, CA).

**Expression constructs**

The cDNAs of mouse Phldb2 (GenBank accession number BC060683) and mouse PSD-95 (GenBank accession number BC 014807) were cloned from Open Biosystems (Open Biosystems, Tokyo, Japan) and were subcloned into a pCAGGS-myc or pCAGGS-FLAG vector. For Phldb2, the primers used were 5'-GAATTCAGATCTATGGCAGAAGATAGCCAC-3' and 5'- GAATTCGTCGACCTACAACAGGAAGTGGGT-3'. For Phldb2ΔPH (deletion at 1189-1296 a.a.), the primers used were 5'-TATACCCACTTCCTGTTGTAG-3' and 5'-TGACACATGAAAACAGGTGTCAAT-3'. For PSD-95, the primers used were 5'-ATCTCGAGATGGACTGTCTCTGTATAGTGACA-3' and 5'-CAGAATTCGGGAGTCTCTCTCGGGCTGGGACCCA-3'. The expression vector for photoactivatable green fluorescent protein-tagged PSD-95 (PAGFP-PSD-95) was a gift from Prof. B.L. Sabatini[^37^](#_ENREF_4), and the EGFP-GluA2 expression vectors were from Prof. J.A. Esteban[^9^](#_ENREF_4).

**Photoactivation**

We performed the photoactivation experiments with PAGFP at synapses^37^. Photoactivation was performed 48 hr after transfection with PAGFP-PSD-95 and tdTomato expression vectors at DIV 21. Images of tdTomato and PAGFP-PSD-95 were obtained with laser excitation at 561 nm and 488 nm. Three iterations of the photoactivation were performed at 5% of the available 730 nm laser power (LSM 710 NLO).The parameters of activation were adjusted within this range to provide near total activity of PAGFP fluorescence while minimizing tdTomato bleaching. Time = 0 image acquisition followed directly after activation. Images were monitored every 30 sec for 30 min using the LSM software ZEN 2009. To analyse the decrease in PAGFP fluorescence, the percentages of fluorescence were calculated as follows: (F– F_i_/ F_0_ – F_i_) x 100. The data were obtained from 10 spines of the *Phldb2^+/+^* mice and 8 spines of the *Phldb2^-/-^* mice.

**BDNF stimulation**

Primary hippocampal neuronal cultures were performed at E17.5. Neurons from *Phldb2^-/-^* mice were plated on a polyethyleneimine-coated 35-mm glass bottom culture dish with an 18-mm central dish (MatTek) at 1×10^4^ cells/central dish, then cotransfected with 0.4 μg of myc-Phldb2 and tdTomato expression vectors were using Lipofectamine 2000 (Invitrogen) at DIV 18. At DIV 19, neurons were cultured in serum-free B27 (Gibco, NY, USA) /Neurobasal medium (Gibco) for 6 hr, and 100 ng/ml BDNF (Sigma-Aldrich) was then added for 1 hr. The cells were observed for that period, and then the BDNF was washed out. B27 supplement, which contains insulin, doesn’t contain the BDNF,

**Hippocampal slice preparation for electrophysiology**

Hippocampal slices were prepared from male *Phldb2^+/+^* mice or *Phldb2^-/-^* mice. The mice were maintained according to the guidelines of the Nara Institute of Science and Technology, and all experiments were approved by the Institutional Animal Care and Use Committee. Artificial cerebrospinal fluid (ACSF) consisted of the following: 125 mM NaCl, 2.6 mM KCl, 1.3 mM MgSO_4_7H_2_O, 1.24 mM KH_2_PO_4_, 26 mM NaHCO_3_, 2.4 mM CaCl_2_ and 10 mM D-glucose. Anesthetized animals were transcardially perfused with ice-cold ACSF to drain the blood and cool the brain. After each animal was decapitated, the whole brain was removed and immersed in ice-cold (4 °C) ACSF bubbled with a mixture of 95% O_2_ and 5% CO_2_. Transverse hippocampus slices (400 µm thick) were prepared using a slicer (LinearSlicer Pro7, Dosaka). The slices were incubated in ACSF at 30 °C for 30 min and then maintained at room temperature for at least 120 min before experimentation. Each slice was transferred to a recording chamber, where it was placed on nylon nets and perfused continuously with oxygenated ACSF at a flow rate of 1.5-2 ml/min. The bath temperature was maintained at 28 °C.

**Electrophysiology**

For electrophysiology, a glass microelectrode (Narishige, Tokyo, Japan) filled with ACSF (2–4 MΏ electrical resistance) was used. fEPSPs were recorded in the stratum radiatum of area CA1 with the glass microelectrode. Experiments were performed according to the methods described with some modifications[^50^](#_ENREF_5). Extracellular stimulation of the Schaffer collateral pathway was accomplished with a nickel-chromium bipolar stimulating electrode (40-µm diameter, Unique Medical, Tokyo, Japan) placed on either side of a CA3 single recording electrode in the stratum radiatum. Evoked fEPSPs were amplified (ER-1 amplifier, Cygnus Technology, Inc. Delaware Water Gap, PA), digitized (DigiData 1200 Interface, Molecular Devices, Palo Alto, CA) and analysed using a program created by Anderson and Collingridge[^55^](#_ENREF_5). The test stimulus intensity was adjusted to produce baseline fEPSP sizes that were 50% of the maximum evoked fEPSP amplitude using an SIU-91 constant current isolator (Cygnus Technology, Southport, NC). Test stimuli were delivered once per minute (0.2 msec stimulus duration) to the Schaffer collaterals. We induced LTP with 100 pulses applied at a rate of 100 Hz for 1 sec and LTD with 900 pulses applied at a rate of 1 Hz for 15 min.

**Input-output relationship and paired-pulse facilitation**

The input-output curve of fEPSP slope (mV/ms) versus presynaptic fibre volleys (FV; mV) at the Schaffer collateral pathway was observed in slices. Paired-pulse facilitation, the short-term enhancement of synaptic efficacy following delivery of two closely spaced stimuli (inter-pulse interval; 25-500 msec), was also assessed.

**T-maze left-right discrimination test**

The left-right discrimination test was conducted using an automatic T-maze (O’Hara & Co., Tokyo, Japan) as previously described^51^. One week before pre-training, mice were deprived of food until their body weight was reduced to 80–85% of the initial level. The mice were kept on a maintenance diet throughout the whole T-maze experiment. Before the first trial, mice were subjected to 30-minute adaptation sessions, during which they were allowed to freely explore the T-maze with all doors open and six locations containing sucrose pellets (pieces of Froot Loop cereal; Kellogg’s, Battle Creek, MI). Starting the day after the adaptation session, the mice were subjected to daily pre-training to introduce them to the food tray. Then, the mice were subjected to a left-right discrimination protocol. One session consisted of 10 trials per day (cutoff time, 50 min). A sucrose pellet was always delivered to the food tray of one of the arms, namely, the goal arm. The mice had to learn to enter the goal arm. *Phldb2^+/+^* mice were trained daily to reach a group average of 80% correct responses in a session. The group average of response accuracy was calculated by averaging the percentage of correct responses of each mouse in a given session and group.

**Twenty four-hour locomotor activity**

We used a system that automatically analysed the locomotor activity of mice in the home cage (15 x 25 x 30 cm) (O’Hara & Co). The animal’s movement was recorded using an electronic monitoring system. The total distance travelled was measured. The animals were placed into the boxes at 8:00 A.M. and were kept there for a 24-hr period. The room light was on from 8:00 A.M. to 8:00 P.M. and was switched off automatically from 8:00 P.M. to 8:00 A.M.; this corresponds exactly with the animals’ normal light/dark cycle in the holding rooms.

**References**

52 Dunkley, P. R., Jarvie, P. E. & Robinson, P. J. A rapid Percoll gradient procedure for preparation of synaptosomes. *Nat Protoc* **3**, 1718-1728, doi:10.1038/nprot.2008.171 (2008).

53 Osten, P. *et al.* The AMPA receptor GluR2 C terminus can mediate a reversible, ATP-dependent interaction with NSF and alpha- and beta-SNAPs. *Neuron* **21**, 99-110 (1998).

54 Vissavajjhala, P. *et al.* Synaptic distribution of the AMPA-GluR2 subunit and its colocalization with calcium-binding proteins in rat cerebral cortex: an immunohistochemical study using a GluR2-specific monoclonal antibody. *Exp Neurol* **142**, 296-312, doi:10.1006/exnr.1996.0199 (1996).

55 Collingridge, G. L., Peineau, S., Howland, J. G. & Wang, Y. T. Long-term depression in the CNS. *Nat Rev Neurosci* **11**, 459-473, doi:10.1038/nrn2867 (2010).

**Supplementary figure legends**

**Supplementary Figure 1.** Phldb2 is expressed in the hippocampus and is localized in the dendritic spine. (**A**) *In situ* hybridisation of Phldb2 mRNA in the brain at P28. The signals were detected with the Phldb2 probe (right panels). The probe with Phldb2 sense sequence was used as a control. Expression of Phldb2 mRNAs was noticed in the hippocampus and the dentate gyrus, especially in pyramidal cells and granule cells, respectively (right panels). Small panels are high-magnified images of the rectangles shown in the upper panels. (**B**) Phldb2 expression was analysed by western blotting using the hippocampi from animals at postnatal day 27. GAPDH was used as an internal control. (**C**) Fluorescent immunohistochemical staining of Phldb2 (green) and NeuN (red) in the brain at 6 weeks. Phldb2 expression was noticed in CA3 of the hippocampus. Scale bars represents 200 μm. (**D**) Expression of Phldb2 and PSD-95 was detected in the hippocampus of 56-day-old mice, especially in the synaptosome and synaptic membrane fractions. (**E**) Immunostaining of Phldb2 (green) and PSD-95 (red) in DIV 21 hippocampal neurons of the *Phldb2^+/+^* mice. Endogenous Phldb2 was co-localized with PSD-95 in the spines. (**F**) Hippocampal neurons from *Phldb2^-/-^* mice were transfected with GFP-Phldb2 expression vectors at DIV 18. Scale bar represents 10 μm.

**Supplementary Figure 2.** Generation of Phldb2 knockout mice. (**A**) Phldb2 allele. (**B**) Targeting strategy. The structures of the wild-type (WT) Phldb2 gene and the targeting construct are shown. Using a genomic clone harbouring the Phldb2 gene, a targeting construct containing exon 2 flanked by loxP sites and the neomycin resistance gene (NEO) flanked by frt sites were generated. Diphtheria toxin A fragment gene (DT-A) was used for negative selection for homologous recombinants. Following homologous recombination in embryonic stem cells, targeted heterozygous mice containing the targeted allele were obtained. Mice harbouring the Phldb2 exon 2-deleted allele were generated by breeding the mice with TNAP-Cre transgenic mice. (**C**) Genomic PCR to detect the WT alleles (*Phldb2^+/+^*) and targeted allele (*Phldb2^+/neo^*) from tail DNA of targeted mice. (**D**) Genomic PCR to detect the WT alleles and Cre-recombined deleted alleles (*Phldb2^-/-^*). (**E**), (**F**) The disruption of the gene and the lack of Phldb2 protein expression were confirmed. The knockout efficiency of Phldb2 was evaluated by RT-PCR. No expression of Phldb2 mRNA was found in the *Phldb2^-/-^* mice. β-actin was used as an internal control. Expression of Phldb2 was analysed by western blotting using cultured hippocampal neurons from *Phldb2^+/+^* mice and *Phldb2^-/-^* mice at DIV 21. GAPDH was used as an internal control in **F**. (**G**) Expression of Phldb2 was detected by immunohistochemistry in the hippocampus CA3 of 6-week-old *Phldb2^+/+^* mice but was not detected in *Phldb2^-/-^* mice of the same age.

**Supplementary Figure 3.** Full blots for data in Figure 2A.

**Supplementary Figure 4.** Phldb2 interacts with PSD-95 and Phldb2 expression rescues the PSD localization in spine of the *Phldb2^-/-^* mice. (**A**) Endogenous PSD-95 was co-immunoprecipitated with Phldb2 in the hippocampi from animals at postnatal day30. Normal IgG was used as negative control. (**B**) For rescue experiments, hippocampal neurons of the *Phldb2^-/-^* mice were transfected with PSD-95-mCherry, GFP expression vectors and myc-Phldb2 or myc expression vector (control). Neurons were fixed at DIV 21. High-magnification image of a mushroom spine. As Fig. 2 **B**, **C**, the spine head region along the red line was divided into 10 parts, shown on the X-axis. The fluorescence peak of PSD-95 was closer to the post synaptic membrane in the *Phldb2*^-/-^ mice with exogenous Phldb2 (n = 11 spines) compared with the *Phldb2^-/-^* mice without exogenous Phldb2 (n = 10 spines). (Mean ± SEM. Student's *t*-test, * *P* < 0.05, Student's *t*-test, * *P* < 0.05).

**Supplementary Figure 5.** The BDNF enhances Phldb2 translocation to spines. (**A**) Cultured hippocampal neurons from *Phldb2^-/-^* mice were cotransfected with expression vectors for GFP-Phldb2 and tdTomato at DIV 18. Neurons were cultured in serum-free B27/neurobasal medium for 6 hr, and 100 ng/ml BDNF was then added for 1 hr at DIV 19. After stimulation of the cells by BDNF, Phldb2 moved to the spines (arrowheads). Scale bar represents 5 μm. (**B**) The LY294002 induced a decrease in numbers of spines with Phldb2-positive heads (Fig. 1A lower row).

**Supplementary Figure 6.** Phldb2 regulates the interaction between NMDA receptor and CaMKII. The interaction between CaMKIIα and the NMDA receptor was analysed by western blotting with cultured hippocampal neurons at DIV 22. Lysates were subjected to immunoprecipitation with anti-CaMKIIα antibody. NMDA receptor subunit NR2A, NR2B or NR1 was co-immunoprecipitated with CaMKIIα in the *Phldb2^+/+^* mice, whereas NMDA receptor subunits were barely detectable in the *Phldb2^-/-^* mice.

**Supplementary Figure 7.** Full blots for data in Figure 4A.

**Supplementary Figure 8.** Separated colour images of Figure 4B, C.

**Supplementary Figure 9.** Phldb2ΔPH expression reduces the surface localization of GluA2. Cultured hippocampal neurons were cotransfected with expression vectors for HA-GluA2, tdTomato and myc-mock (control) (**A**) or with expression vectors for HA-GluA2, tdTomato and myc-Phldb2ΔPH (**B**) at DIV 19. First, the neurons were stained for surface HA-GluA2 (green). Subsequently, after the Triton X-100 treatment, the neurons were subjected to HA-GluA2 staining (total GluA2, shown in blue). The dendritic regions shown in squares are magnified in the bottom panels (arrowheads). (**C**) The fluorescence intensity of the surface GluA2 was divided by that of total GluA2 (surface/total ratio of GluA2) in the myc-mock expression and was defined as 1.0 for normalization. The normalized surface/total GluA2 ratio was decreased in cells expressing Phldb2ΔPH (Mean ± SEM. Student's *t*-test,** *P* < 0.01). Scale bars = 20 μm.

**
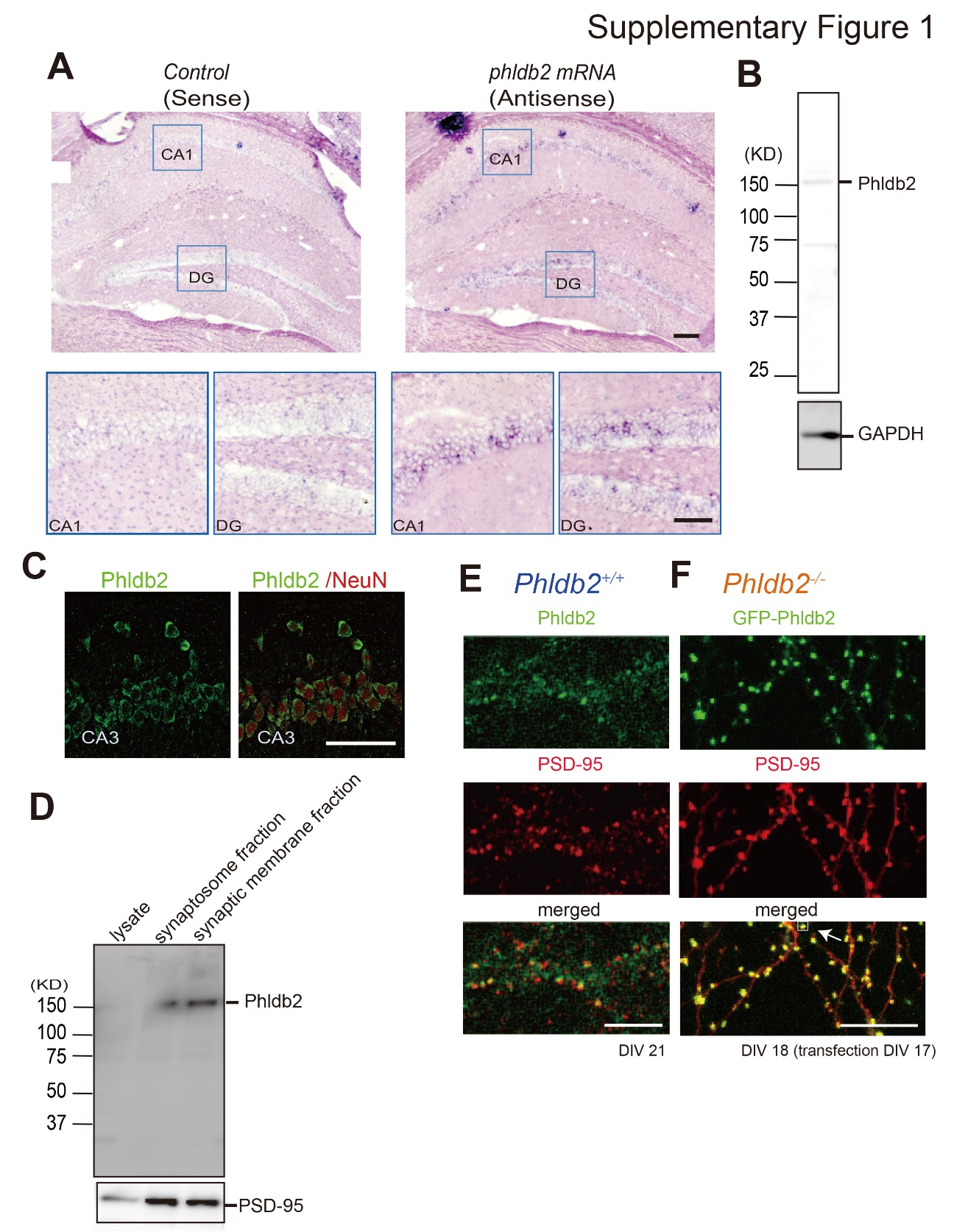

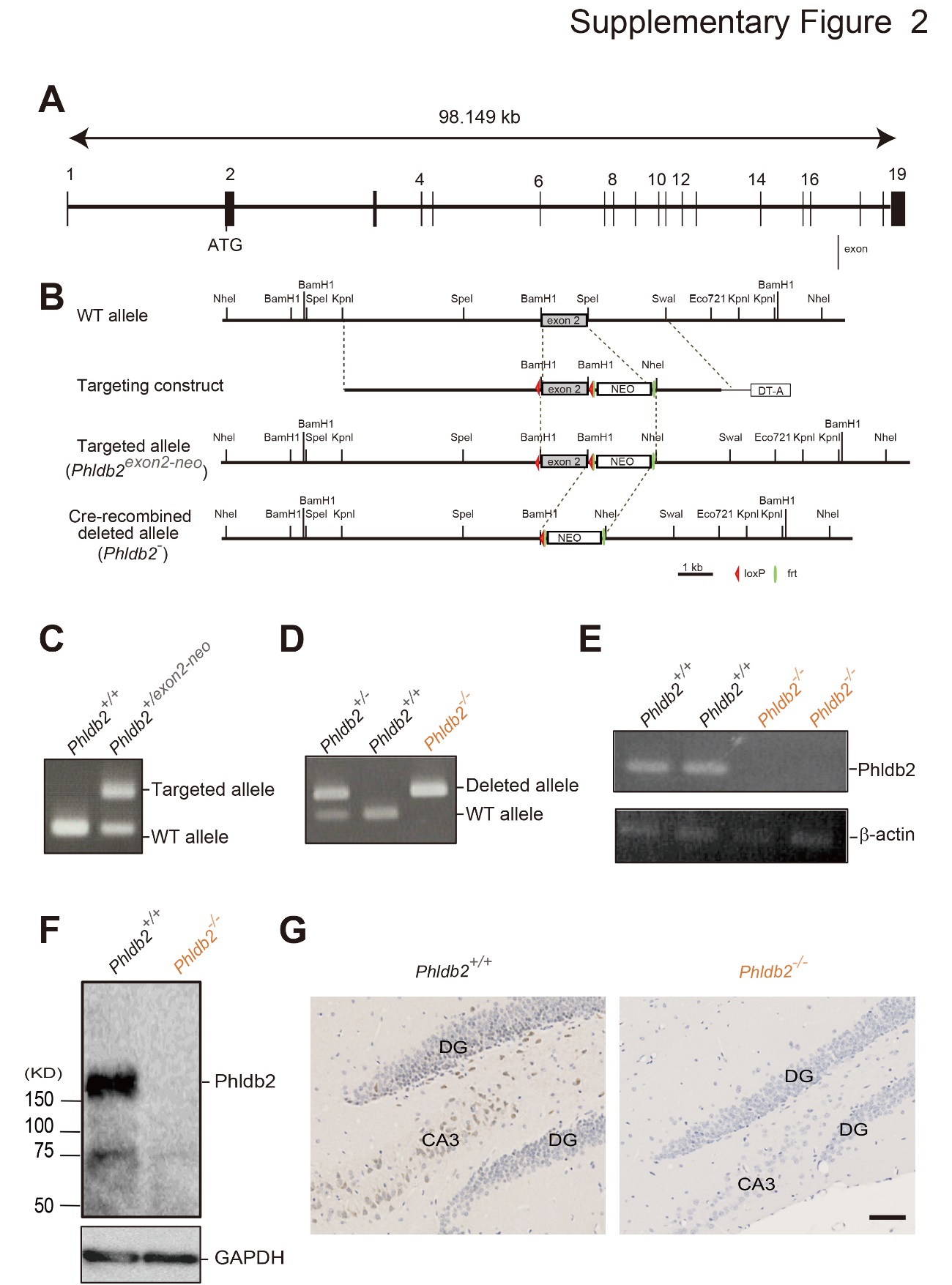

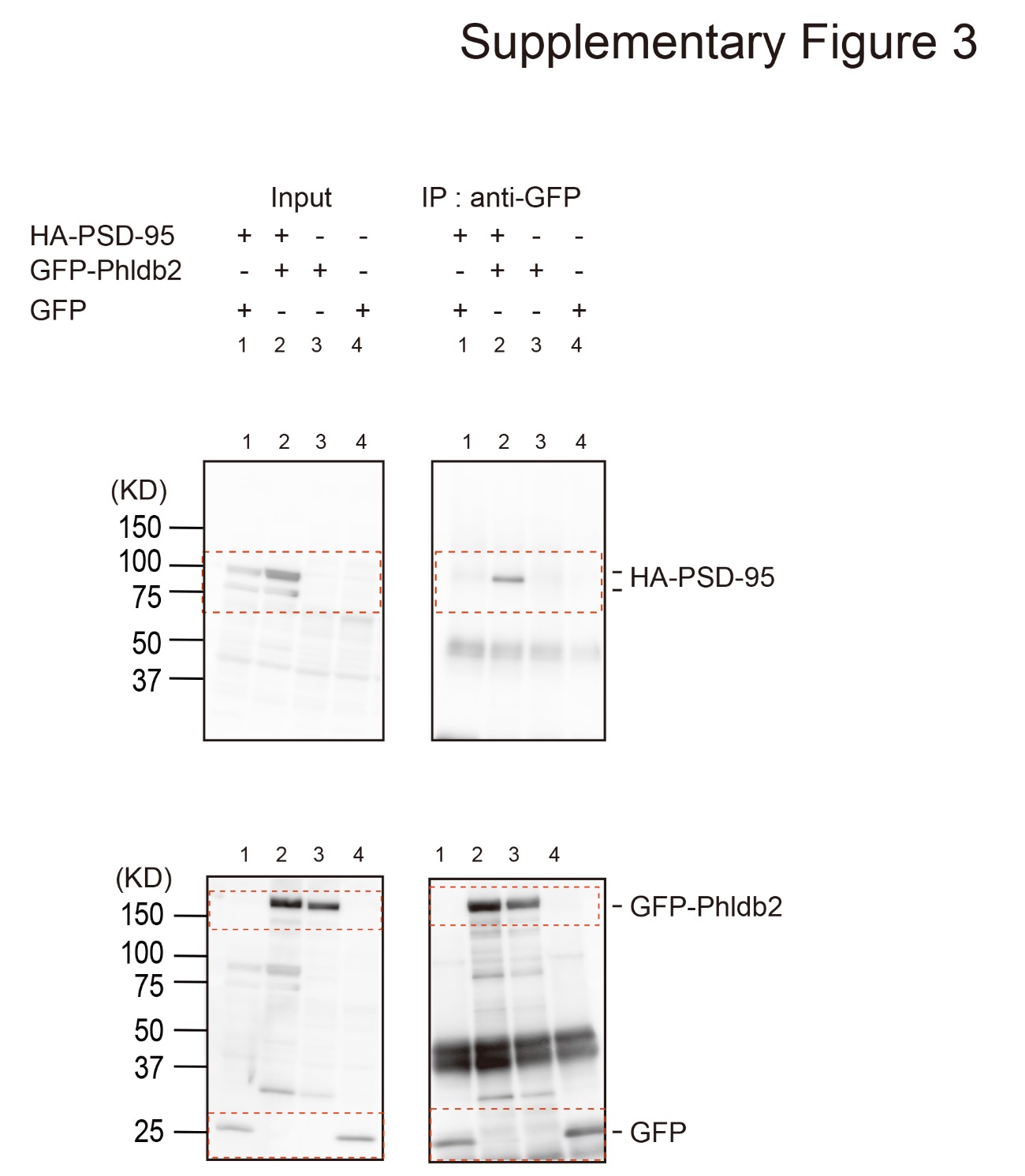

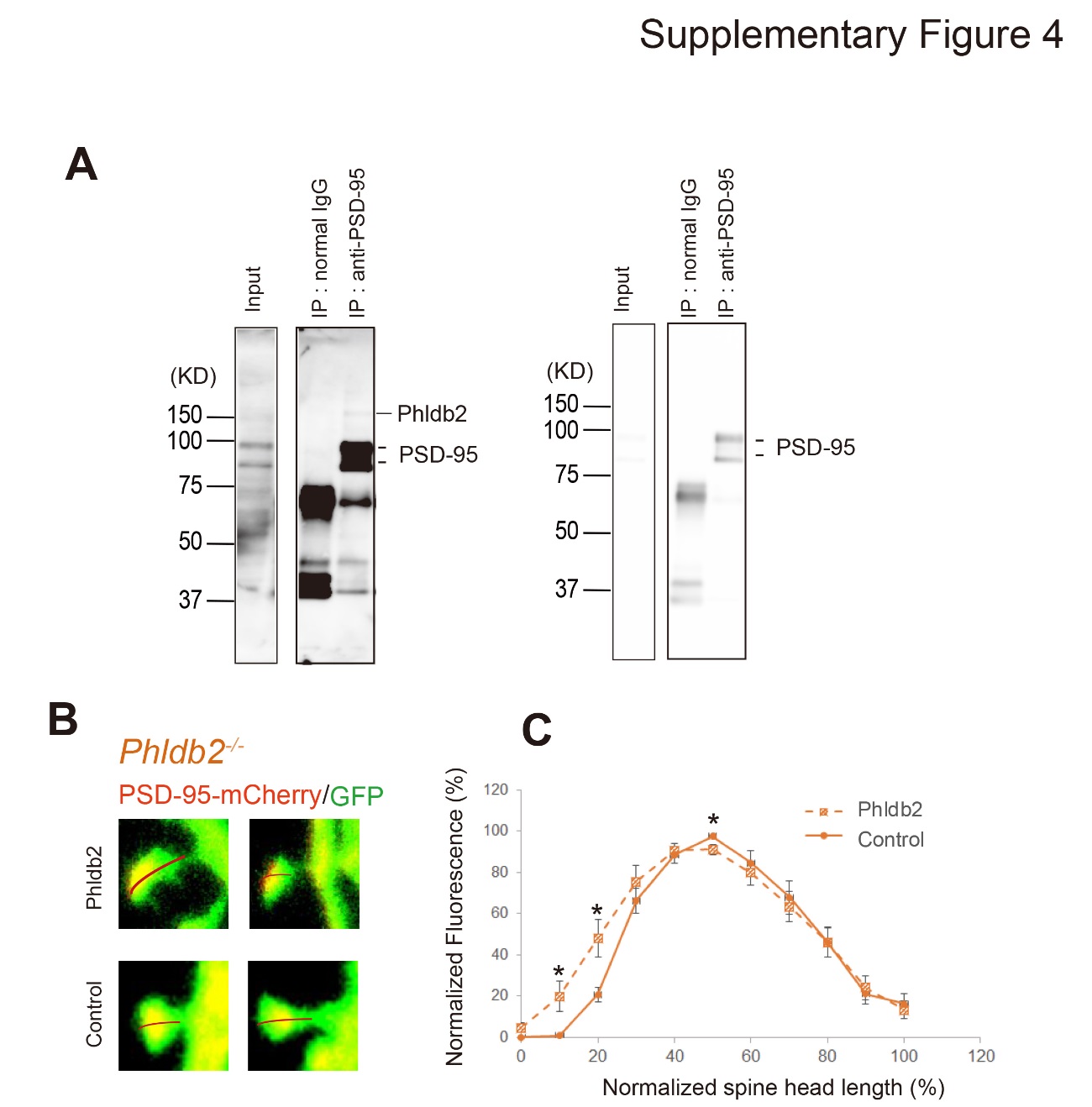

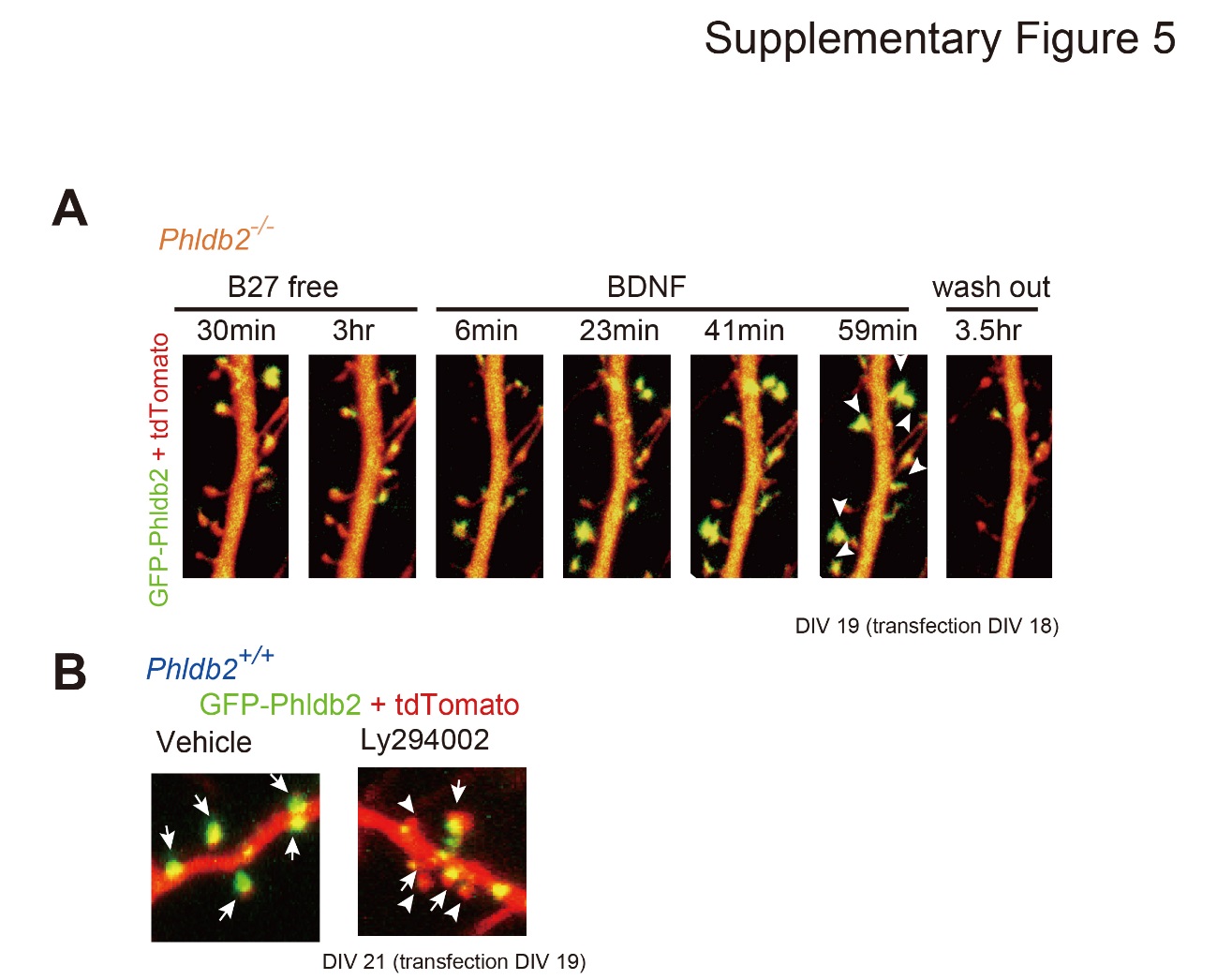

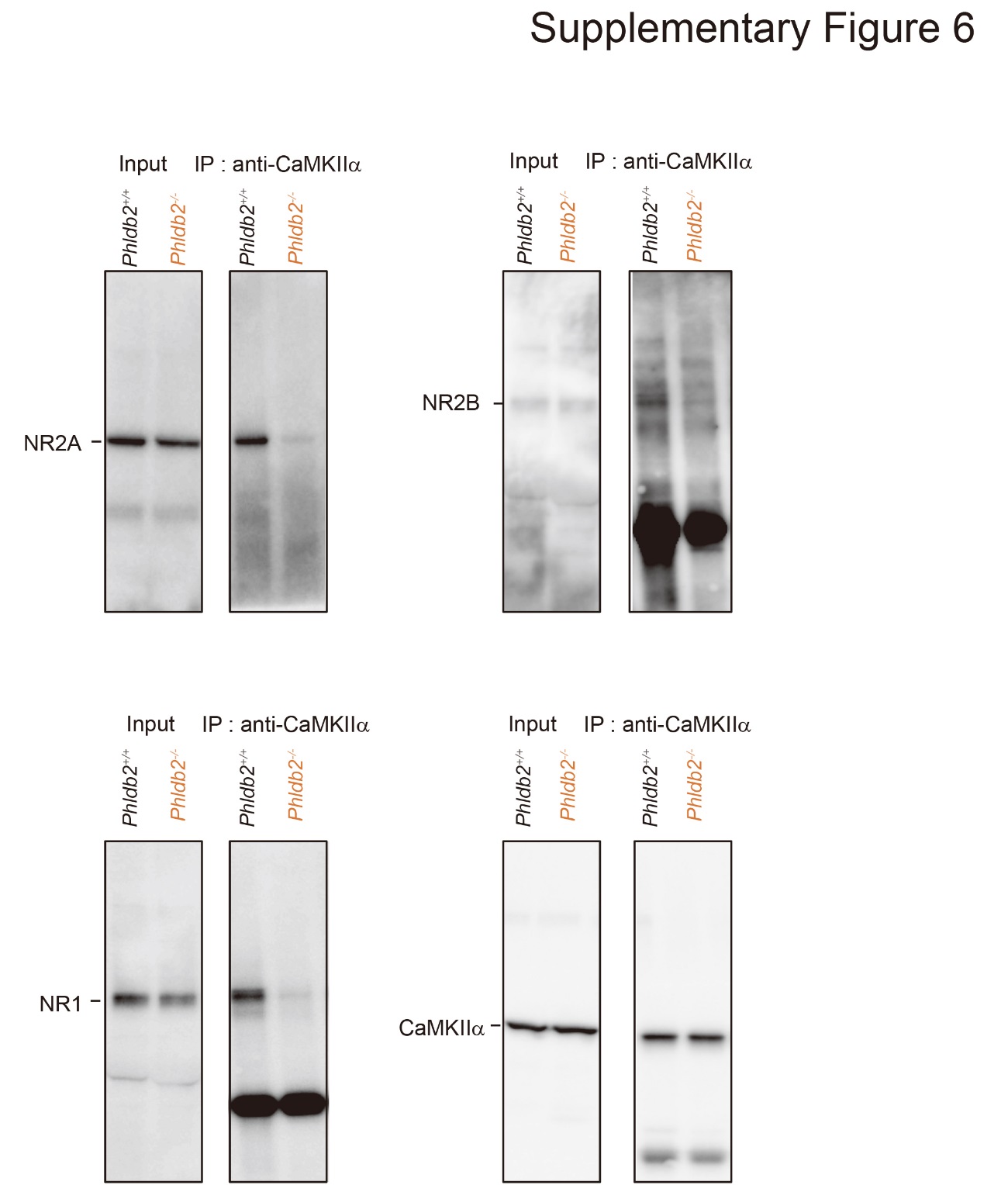

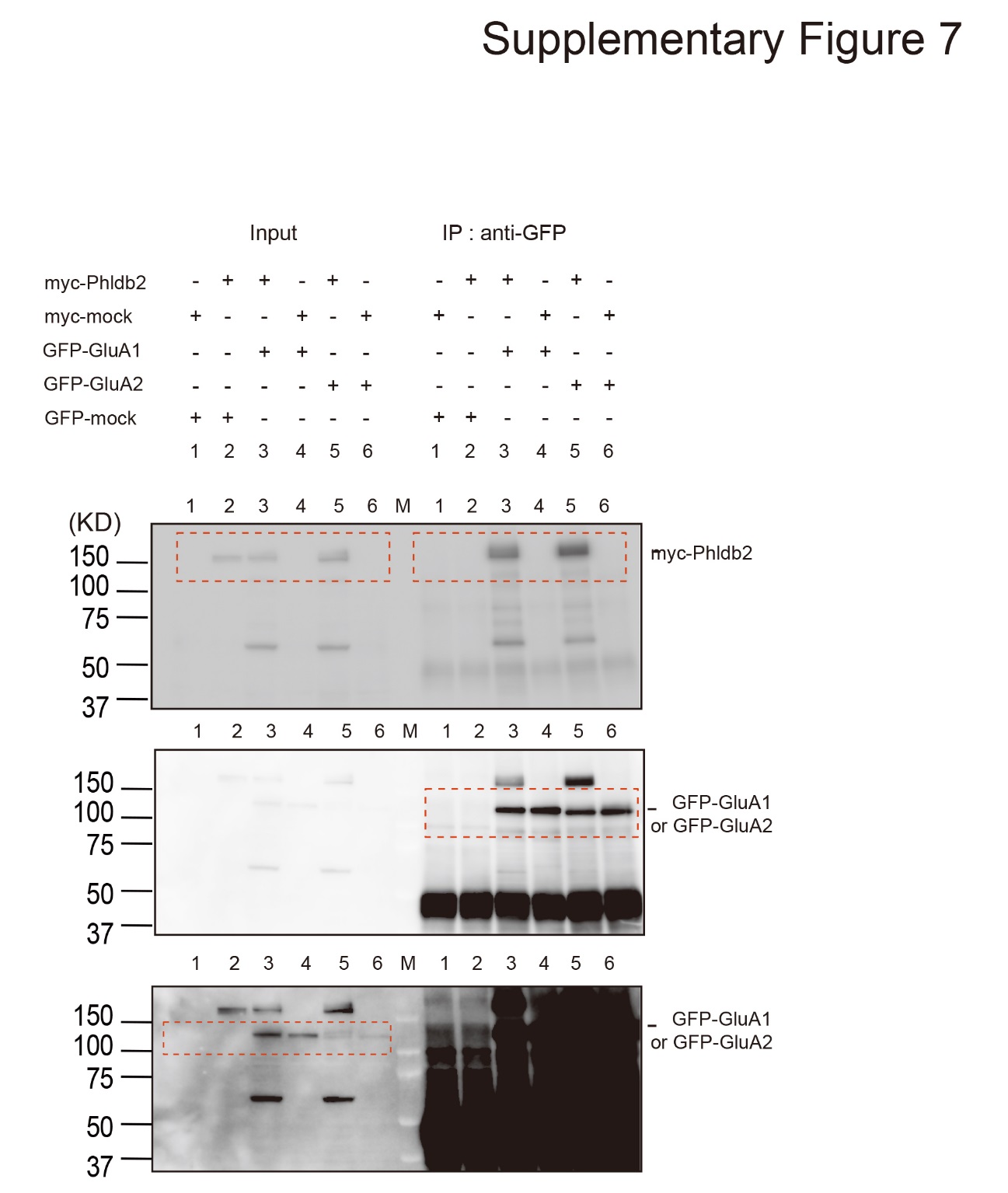

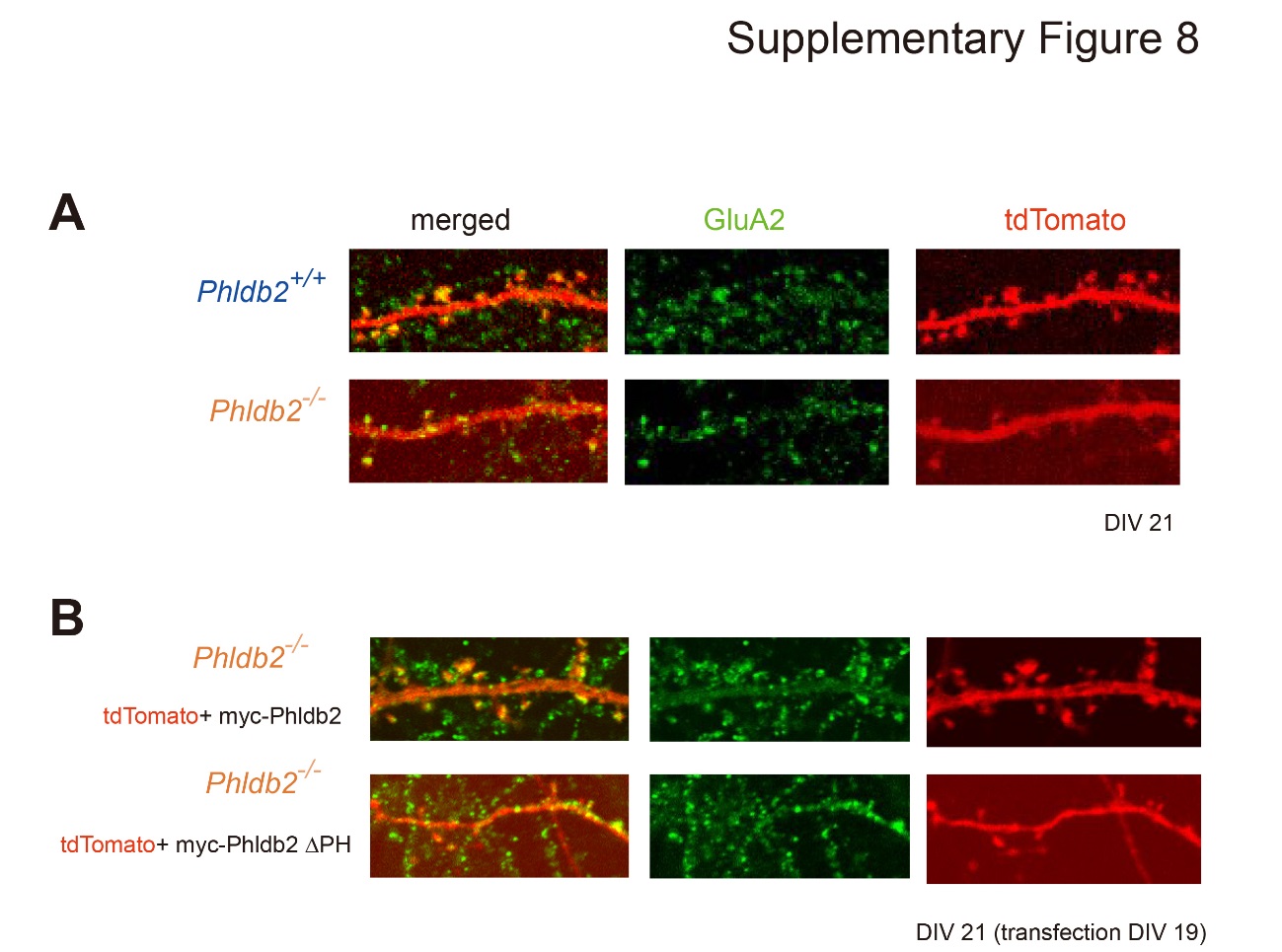

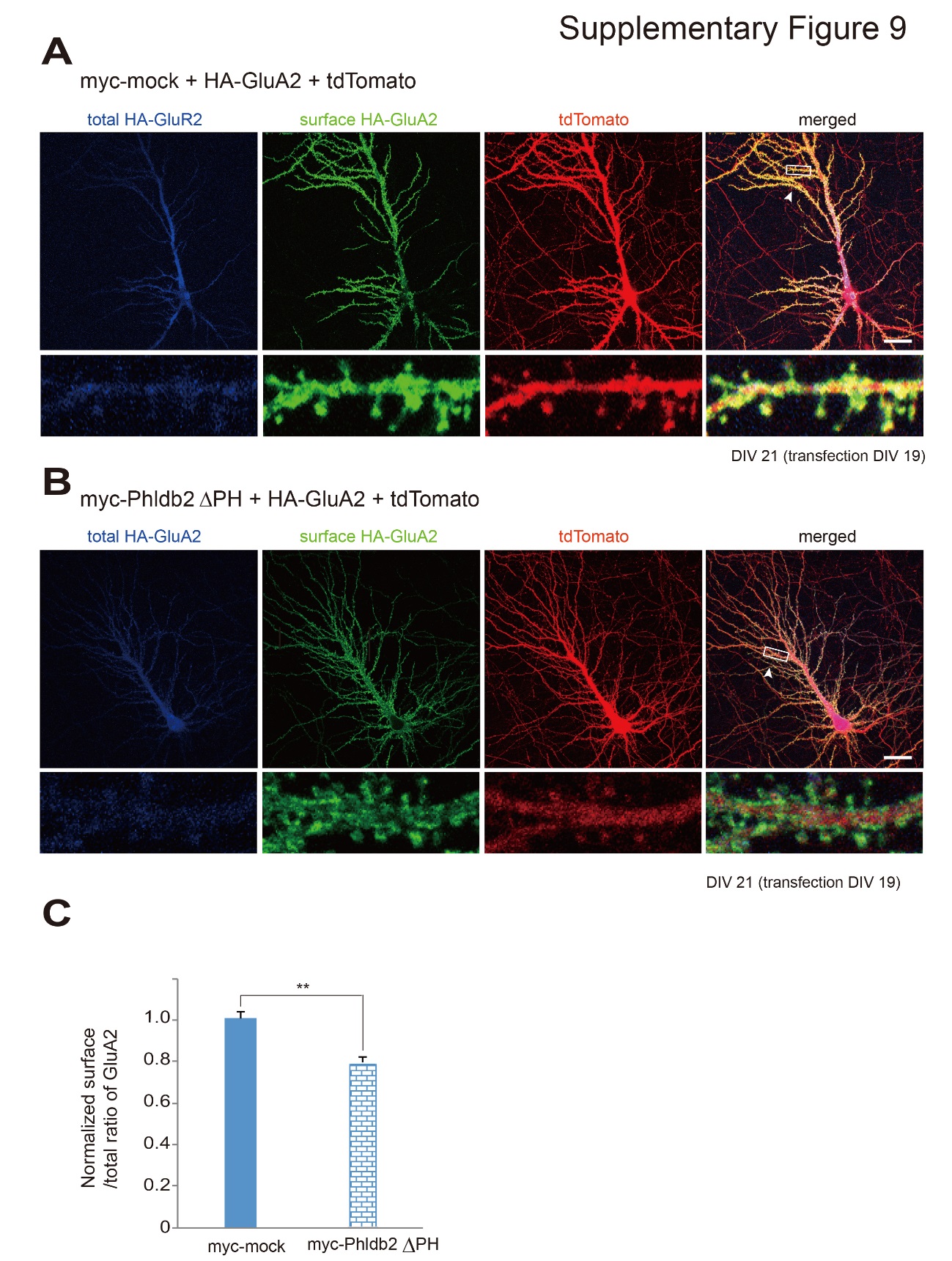
**
